# Genotoxic and Physiological Responses to Repeated Exposure to Low-Concentration Silver Nanoparticles in *Vicia faba* Through Root and Foliar Uptake Pathways

**DOI:** 10.3390/ijms27177733

**Published:** 2026-08-28

**Authors:** Paulina Abrica-González, Norberto Alarcón-Herrera, Sandra Gómez-Arroyo, Saúl Flores-Maya, José Abraham Balderas-López, Edgar A. Estrella-Parra

**Affiliations:** 1 Laboratorio de Técnicas Fototérmicas, Unidad Profesional Interdisciplinaria de Biotecnología, Instituto Politécnico Nacional, Av. Acueducto, La Laguna Ticomán, Gustavo A. Madero, Mexico City 07340, Mexico; 2 Laboratorio de Genotoxicología Ambiental, Instituto de Ciencias de la Atmósfera y Cambio Climático, Universidad Nacional Autónoma de México, Ciudad Universitaria, Coyoacán, Mexico City 04510, Mexico; 3 Laboratorio de Fitoquímica, UBIPRO, Facultad de Estudios Superiores (FES) Iztacala, Universidad Nacional Autónoma de México, Av. de los Barrios 1, Los Reyes Iztacala, Tlalnepantla 54090, Estado de México, Mexico; norberto.alarconh@gmail.com (N.A.-H.);; 4 Laboratorio de Recursos Naturales, UBIPRO, Facultad de Estudios Superiores (FES) Iztacala, Universidad Nacional Autónoma de México, Av. de los Barrios 1, Los Reyes Iztacala, Tlalnepantla 54090, Estado de México, Mexico

**Keywords:** comet assay, micronucleus, biomarkers, nanomaterial characterization, nanotoxicity, cytogenetic

## Abstract

Silver nanoparticles (AgNPs) are among the most widely used engineered nanomaterials, increasing the likelihood of repeated plant exposure through soil, water, and atmospheric deposition. However, the biological consequences of repeated exposure through different uptake pathways remain insufficiently understood. This study characterized citrate-reduced AgNPs and evaluated their genotoxic and physiological effects in *Vicia faba* following repeated root and foliar exposure. Physicochemical characterization included SEM, energy-dispersive X-Ray spectroscopy, dynamic light scattering, zeta potential analysis, and UV–Visible spectroscopy. Cytogenetic damage was assessed using the mitotic index, micronucleus test, chromosomal and nuclear abnormalities, and alkaline comet assay, while chlorophyll content and stem growth were determined as physiological endpoints. The synthesized AgNPs were predominantly spherical, exhibited colloidal stability, and produced greater biological effects than bulk silver. Repeated exposure reduced cell proliferation and increased micronuclei, chromosomal aberrations, nuclear buds, binucleated cells, and DNA damage in both roots and leaves. Root tissues consistently showed greater DNA damage following AgNP exposure, while AgNPs also caused greater reductions in chlorophyll content and plant growth than bulk silver. These findings demonstrate that repeated AgNP exposure induces tissue-specific and persistent biological responses, emphasizing the importance of incorporating repeated-exposure scenarios and multiple uptake pathways into environmental risk assessments of engineered nanomaterials.

## 1. Introduction

Nanotechnology has emerged as a rapidly expanding discipline, advancing the development of materials with distinctive properties originating from their nanoscale dimensions. Among these materials, engineered nanoparticles are distinguished by their high surface area to volume ratio and unique physicochemical characteristics, which impart catalytic, optical, electrical, and biological properties that markedly differ from those of their bulk counterparts. These attributes have facilitated their extensive integration into a variety of applications, including biomedical sciences, electronics, agriculture, the food industry, and environmental remediation [1,2,3,4]. However, the continuous increase in the production and use of these nanomaterials has also increased the likelihood of their environmental release, raising concerns about their environmental fate and potential effects on non-target organisms [5,6]. Consequently, the assessment of risks associated with nanoparticles has become a priority in ecotoxicological research [7].

Among metallic nanoparticles, AgNPs are among the most extensively produced and commercially used nanomaterials worldwide. Their widespread application is primarily due to their well-documented antimicrobial properties, which have enabled their incorporation into a broad range of products, including medical devices, wound dressings, antimicrobial textiles, personal care products, food packaging materials, paints, detergents, household appliances, and water purification systems [8,9,10]. The continuous growth of the market for AgNPs containing products has significantly increased the likelihood of environmental release of these nanoparticles throughout their life cycle, including production, use, washing, wear, and final disposal [10,11].

Multiple studies have demonstrated that AgNPs may be released into aquatic and atmospheric environments via industrial effluents, domestic wastewater, landfill leachates, sewage sludge, and aerosol emissions [12,13]. Wastewater treatment facilities have been recognized as a significant conduit for the ingress of AgNPs into the environment, owing to the limited efficacy of traditional treatment methods in eliminating these nanomaterials. As a result, considerable amounts of AgNPs may be discharged into surface water bodies and subsequently accumulate in sediments and agricultural soils via the application of biosolids [11]. Similarly, the release of nanoparticles into the atmosphere from industrial emissions and the degradation of consumer products promotes their dispersion and subsequent deposition onto terrestrial ecosystems [12,13,14]. Therefore, the large-scale production and continuous release of AgNPs constitute an emerging environmental issue that increases the probability of exposure of numerous organisms, particularly plants, to these contaminants [8,15].

Plants represent one of the initial biological compartments to encounter nanoparticle contamination within terrestrial ecosystems. As primary producers, they hold an indispensable role in maintaining ecosystem functionality and represent a significant pathway for the transfer of contaminants through trophic chains [16,17]. Owing to their sessile nature and direct interactions with soil, water, and atmospheric deposits, plants can absorb, accumulate, and respond to nanomaterials present in the environment [18]. Consequently, plant models have acquired significant importance in ecotoxicological research by offering valuable insights into the effects of emerging contaminants and aiding in the evaluation of environmental risks related to the escalating release of nanoparticles [19,20].

Plant bioassays provide numerous benefits compared to alternative experimental models. These advantages include cost-effectiveness, simplicity of handling, heightened sensitivity, brief exposure durations, and the ability to concurrently assess physiological, cytotoxic, and genotoxic responses [21,22]. Furthermore, plants exhibit highly conserved cellular mechanisms involved in DNA replication and repair, as well as chromosome segregation, rendering them dependable models for the detection of genotoxic agents. The evaluation of genetic damage in plants is of particular significance, as alterations in genome integrity can impact growth, reproduction, productivity, and the stability of ecosystems [23,24].

Among the available plant models, *V. faba* is widely acknowledged as one of the most sensitive and dependable biological systems for assessing environmental cytotoxicity and genotoxicity [25]. The root meristematic cells of this species display features that support cytogenetic analyses, such as a low chromosome number (2n = 12), large sized chromosomes, and a high mitotic index. These characteristics facilitate the precise observation of chromosomal modifications caused by various mutagenic and clastogenic agents [26,27,28]. In addition to root-based assays, leaf tissues of *V. faba* have also been utilized as experimental models to assess physiological and biochemical responses. These include modifications in photosynthetic pigments, markers of oxidative stress, and antioxidant defense systems following exposure to environmental contaminants [29,30]. Due to these advantages, *V. faba* has been extensively employed in biomonitoring programs and in the assessment of the toxic effects of heavy metals, pesticides, industrial contaminants, and various nanomaterials [25].

Research has demonstrated the considerable genotoxic potential of silver nanoparticles AgNPs across various biological systems, including microorganisms, mammalian cells, and higher plants. In plant models, AgNPs have been documented to induce cytogenetic alterations, decrease the mitotic index, and elevate the frequency of micronuclei and chromosomal aberrations in *V. faba* [31]. Likewise, studies conducted in mammalian cells have shown that AgNPs can induce DNA damage, chromosomal instability, and impaired cell viability [32,33]. These genotoxic effects have been linked to the production of reactive oxygen species, oxidative damage to DNA, mitochondrial impairment, and the liberation of Ag^+^ ions [34,35].

Nevertheless, most available studies have assessed the effects of AgNPs in the context of acute or single-exposure scenarios, which are unlikely to accurately depict realistic environmental exposure conditions [36]. Given the extensive manufacturing and ongoing dissemination of AgNPs from various consumer goods, these nanomaterials have the potential to remain in aquatic and terrestrial environments, thereby creating scenarios of chronic or repeated exposure to plant organisms. In such circumstances, plants may undergo successive exposures to trace concentrations of nanoparticles, which may lead to the gradual buildup of physiological and genetic impairments that are inadequately evaluated through single-exposure studies [37,38].

Consequently, understanding of the effects resulting from repeated exposure to AgNPs, as well as the potential accumulation of DNA damage, chromosomal alterations, and phytotoxic responses in higher plants, remains limited [36,37]. Furthermore, the relationship between the physicochemical properties of nanoparticles and the magnitude of the induced biological responses remains insufficiently understood [39,40]. Given that attributes such as size, morphology, aggregation state, and surface properties primarily influence the environmental behavior, bioavailability, and toxicity of AgNPs, it is imperative to incorporate the physicochemical characterization of these nanomaterials alongside the assessment of molecular, cytogenetic, and physiological biomarkers under exposure scenarios that more accurately simulate realistic environmental conditions [16,40,41].

Therefore, considering the increasing production and environmental release of silver nanoparticles and the need for sensitive plant models to assess the ecological risks associated with these emerging contaminants, the present study aimed to characterize silver nanoparticles and evaluate their phytotoxic and genotoxic effects in *V. faba* under a repeated exposure regime. This evaluation was conducted through the determination of chlorophyll content, leaf development, DNA damage using the comet assay, and chromosomal aberrations. Genotoxic responses were assessed in two plant structures: roots exposed to nanoparticles in an aqueous medium and leaves exposed through nebulization, in order to emulate the principal environmental routes of nanoparticle entry and provide a more realistic approximation of the exposure scenarios that plants may experience under natural conditions.

## 2. Results

### 2.1. Characterization of Silver Nanoparticles

The synthesized AgNPs were subjected to physicochemical characterization to ascertain their size, size distribution, and surface charge prior to the assessment of their biological effects. The findings are summarized in Table 1.

**Figure 1:**
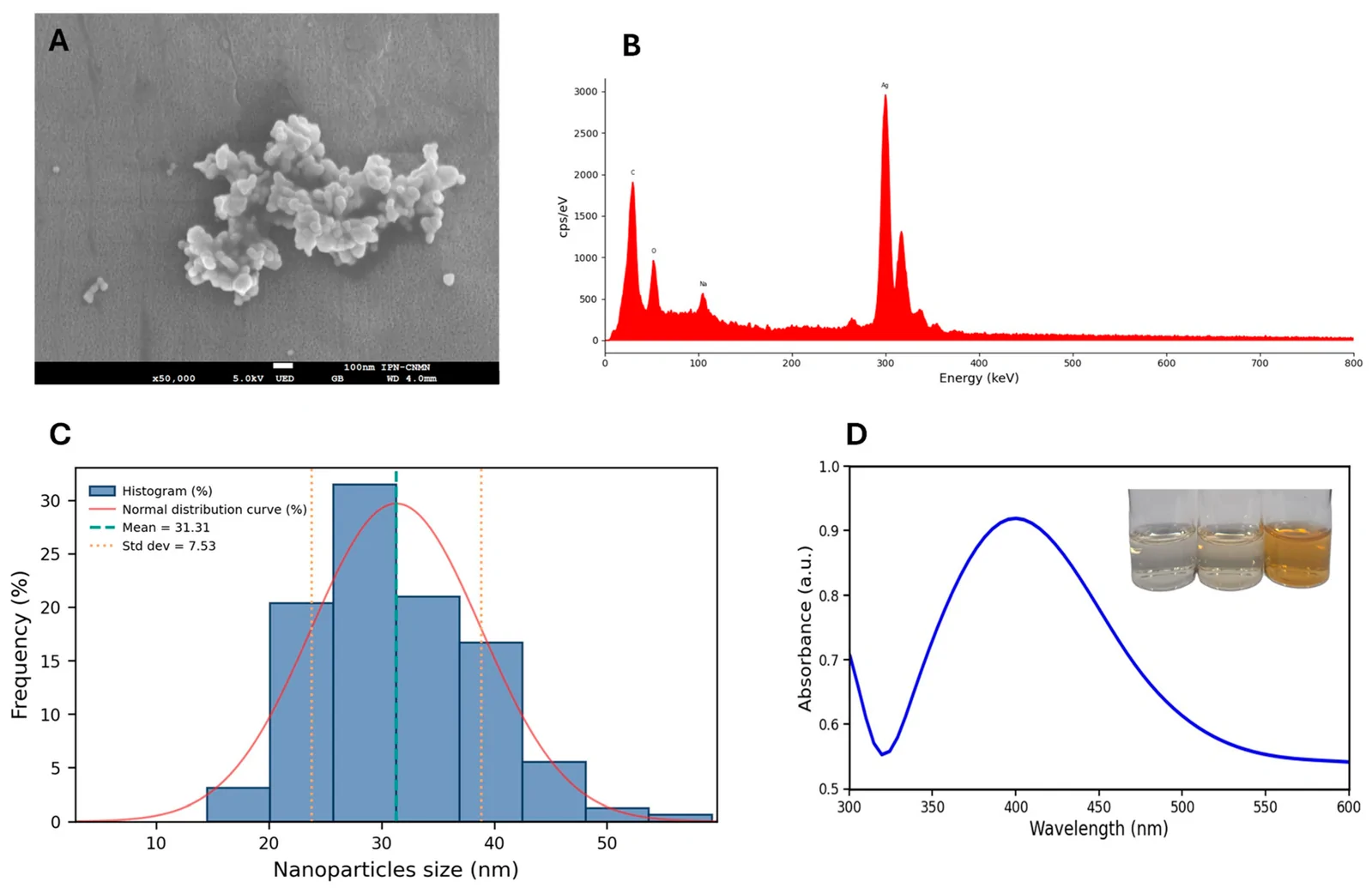
Physicochemical characterization of silver nanoparticles (AgNPs): (**A**) SEM micrograph of the synthesized AgNPs. (**B**) Energy-dispersive X-Ray spectroscopy (EDS) spectrum confirming the elemental composition and the presence of silver. (**C**) Particle size distribution showing an average diameter of 31.31 ± 7.53 nm. (**D**) UV–Vis absorption spectrum displaying the characteristic surface plasmon resonance (SPR) band at 400.23 nm, confirming the successful formation of AgNPs. The inset shows representative photographs of AgNP suspensions at concentrations of 5, 10, and 50 mg/L (**left** to **right**).

Transmission electron microscopy analysis demonstrated that the synthesized silver nanoparticles were predominantly spherical and polyhedral in morphology, with only minimal agglomeration observed among certain particles (Figure 1A and Table 1). The nanoparticles displayed a uniform surface morphology and appeared well dispersed, signifying the successful synthesis of stable nanostructures. EDS (Figure 1B) confirmed the elemental composition of the synthesized silver nanoparticles. The spectrum exhibited a characteristic and intense Ag signals at approximately around of 3.0 keV, corresponding to the Ag Lα emission line, confirming the successful formation of silver nanoparticles. Additional signals were detected at approximately 1.90 keV, 0.96 keV, and 0.56 keV, which were attributed to carbon (C), oxygen (O), and sodium (Na), respectively. The presence of carbon and oxygen signals may be associated with residual organic components and surface bound citrate molecules, while the sodium signal is attributed to the sodium citrate used as the reducing and stabilizing agent during nanoparticle synthesis.

The particle size distribution derived from SEM measurements demonstrated a normal distribution (Figure 1C). AgNPs exhibited a mean diameter of 31.31 ± 7.53 nm, with most particles measuring between 35 and 45 nm. This indicates a narrow size distribution and low polydispersity.

DLS measurements (Table 1) showed a hydrodynamic diameter of 50.94 ± 0.126 nm and a PDI of 0.176 ± 0.004. The zeta potential was −27.9 ± 3.45 mV.

The UV–Visible absorption spectrum of the AgNPs displayed a characteristic surface plasmon resonance (SPR) band with a peak in 400.23 nm (Figure 1D). The presence of this absorption band is indicative of metallic silver nanoparticles and further substantiates the successful synthesis of AgNPs. In combination, SEM, particle size distribution, and UV–Vis analyses confirmed the formation of uniform AgNPs with an average size of approximately 30 nm and demonstrated considerable colloidal stability. Representative photographs of the AgNPs suspensions at concentrations of 5, 10, and 50 mg/mL displayed a progressive enhancement in color intensity correlating with increasing nanoparticle concentration (Inset figure in Figure 1D). The suspensions manifested the characteristic yellowish-brown coloration associated with AgNPs formation, and no visible signs of precipitation or aggregation were observed, indicating good colloidal dispersion across the studied concentration range.

### 2.2. Stability Assays of Silver Nanoparticles

**Figure 2:**
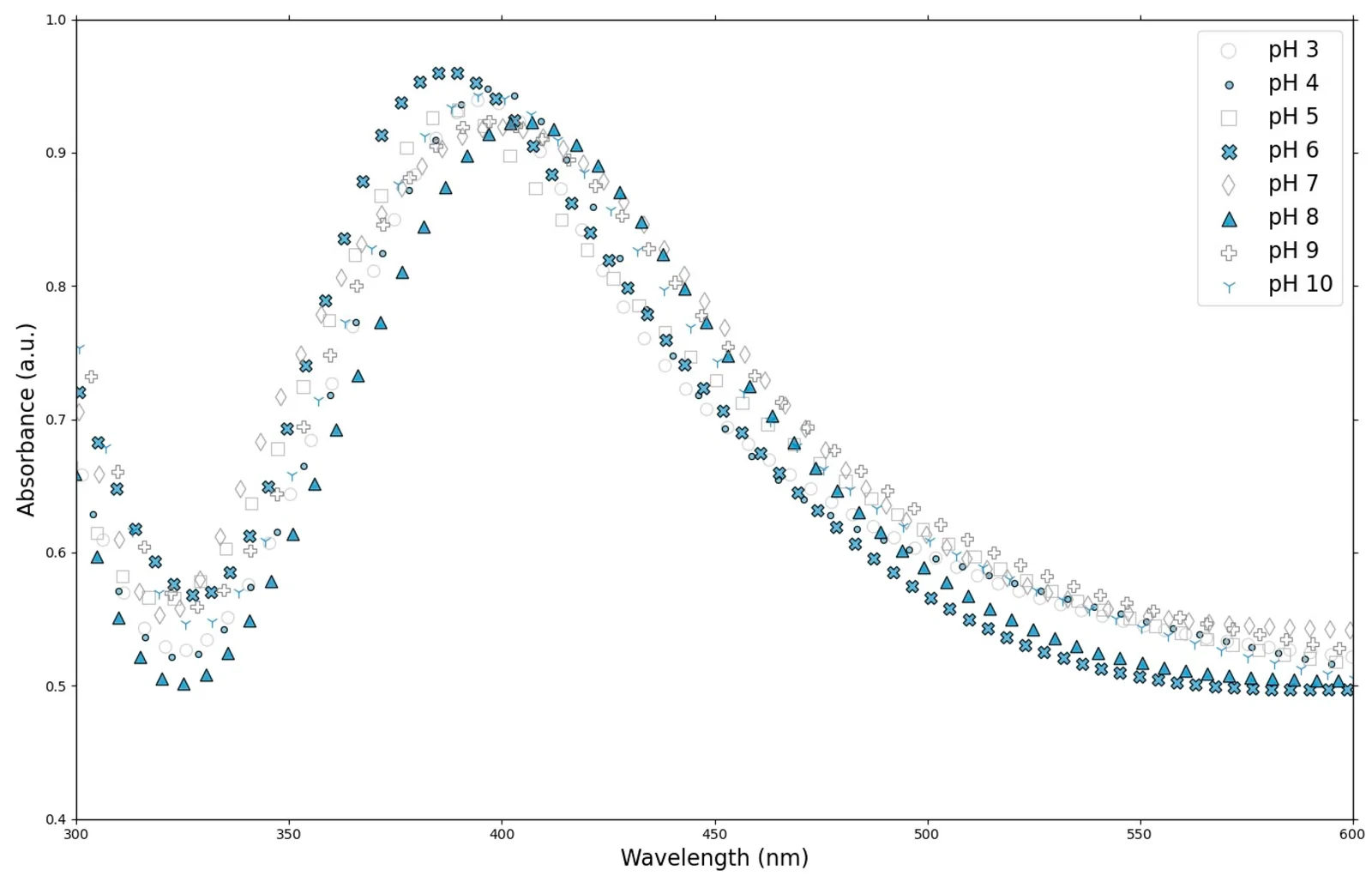
UV–Visible absorption spectra of AgNPs under different pH conditions (pH 3–10). The spectra show a SPR band located between 390 and 410 nm across all evaluated pH values. Slight variations in absorbance intensity and minor changes in band shape are observed among treatments, while the overall spectral profile is maintained throughout the analyzed pH range.

The UV–Visible spectra of AgNPs at different pH conditions (pH 3–10) are depicted in Figure 2. A surface plasmon resonance band is consistently observed within the approximate wavelength range of 390–410 nm across all examined conditions.

Across the different pH treatments, slight variations in absorbance intensity are observed, along with minor changes in band width. The position of the SPR band remains within the same spectral region (390–410 nm) under all tested pH values.

These spectral profiles correspond to AgNPs analyzed under acidic, neutral, and alkaline conditions, indicating that the nanoparticles remain stable across the evaluated pH range.

### 2.3. Chromosomal Aberrations

#### 2.3.1. Mitotic Index

The mitotic index was assessed as a marker of cytotoxicity and cellular proliferative capacity, given that its decrease signifies cell cycle arrest induced by the treatments (Table 2).

One-way analysis of variance (ANOVA) revealed a significant treatment effect on the mitotic index (F = 7.10, *p* = 0.001). Dunnett post hoc test, using the control group as the reference, showed that Ag/B 5, 10, 50, AgNPs 5, and AgNPs 10 significantly reduced the mitotic index compared with the control (*p* < 0.05), whereas AgNPs 50 did not differ significantly from the control (*p* > 0.05).

When evaluating the effect of concentration within each silver form, no significant differences were detected among bulk silver treatments (F = 0.42, *p* = 0.676), a result confirmed by Tukey’s test (*p* > 0.05). Similarly, silver nanoparticles did not show significant differences among concentrations (F = 2.42, *p* = 0.170; Tukey, *p* > 0.05).

Comparisons between bulk and nanoparticulate forms using Student’s *t*-test indicated no significant differences at equivalent concentrations (Ag/B 5 vs. AgNPs 5, *p* = 0.680; Ag/B 10 vs. AgNPs 10, *p* = 0.109; Ag/B 50 vs. AgNPs 50, *p* = 0.172).

The distribution of mitotic phases exhibited significant variation among the different treatments (see Table 1). In the negative control, a predominant proportion of dividing cells were identified in prophase (89.1%), whereas the proportions in metaphase, anaphase, and telophase were 6.9, 2.5, and 1.4%, respectively. Conversely, the positive control, potassium dichromate, resulted in a complete cessation of mitotic progression, with all dividing cells being confined to prophase (100%).

Exposure to bulk silver particles resulted in a more heterogeneous distribution of mitotic phases. At 5, 10, and 50 mg/L, prophase represented approximately 60–64% of mitotic cells, while metaphase ranged from 18–20%, anaphase from 9–12%, and telophase from 3–12%, indicating a partial redistribution of cells across mitotic stages compared with the control.

A similar pattern was observed in AgNPs treated roots, although with more pronounced phase specific shifts. At 5 and 10 mg/L, prophase accounted for 60.5% and 65.7% of dividing cells, respectively, accompanied by increases in metaphase and anaphase frequencies. Notably, exposure to 50 mg/L AgNPs resulted in the highest proportion of cells in anaphase (20.9%), representing an approximately eight fold increase relative to the negative control (2.5%), while telophase frequency decreased to 3.9%.

Overall, both bulk silver particles and AgNPs influenced the progression of the cell cycle in *V. faba*, resulting in a redistribution of cells among mitotic phases and indicating a potential disturbance in normal mitotic progression.

#### 2.3.2. Micronucleus Frequency

**Figure 3:**
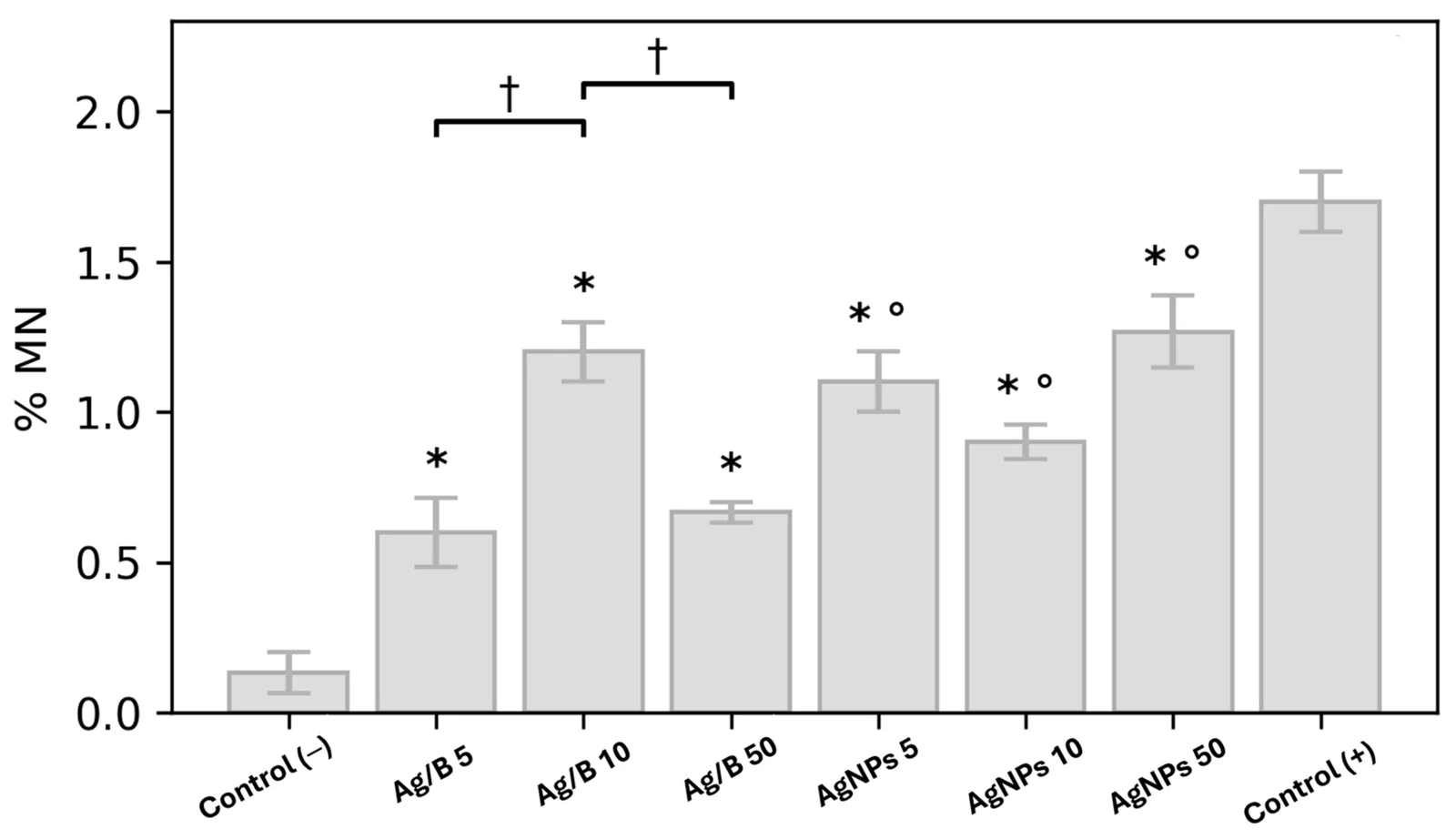
Genotoxicity (%MN) in roots of *V. faba* exposed to Ag/B and AgNPs at concentrations of 5, 10, and 50 mg/L, with potassium dichromate used as the positive control. Data are presented as the mean ± standard error (SE). The frequency of micronuclei (%MN) increased in response to both forms of silver, indicating genotoxic effects that varied according to concentration and particle form. * indicates a significant difference compared with the negative control (*p* < 0.05). † indicate significant differences among concentrations within the same material (Ag/B or AgNPs) (*p* < 0.05). ° indicates a significant difference between bulk silver and AgNPs at the corresponding concentration (*p* < 0.05).

Micronucleus (MN) index (Figure 3) is evaluated as a biomarker of genotoxicity, as its elevation indicates chromosomal breakage or chromosome loss events during cell division.

ANOVA demonstrated a statistically significant influence of the treatment on MN frequency (F = 44.71, *p* < 0.001). Dunnett test indicated that all treatments, encompassing both bulk and nanoparticulate silver, resulted in a significant increase in MN frequency relative to the control group (*p* < 0.05).

In bulk silver treatments, significant differences were observed among concentrations (F = 66.02, *p* < 0.001), with Ag10 showing a higher MN frequency than Ag/B 5 and Ag/B 50 (*p* < 0.05). Conversely, silver nanoparticles did not demonstrate a dose-dependent effect (F = 3.64, *p* = 0.092), with no statistically significant differences among AgNPs 5, AgNPs 10, and AgNPs 50 (*p* > 0.05).

Comparisons between silver forms revealed differential responses between bulk and nanoparticles at all concentrations (Ag/B 5 vs. AgNPs 5, *p* = 0.047; Ag/B 10 vs. AgNPs 10, *p* = 0.016; Ag/B 50 vs. AgNPs 50, *p* = 0.041).

#### 2.3.3. Chromosomal Aberrations, Nuclear Buds and Binucleated Cells

**Figure 4:**
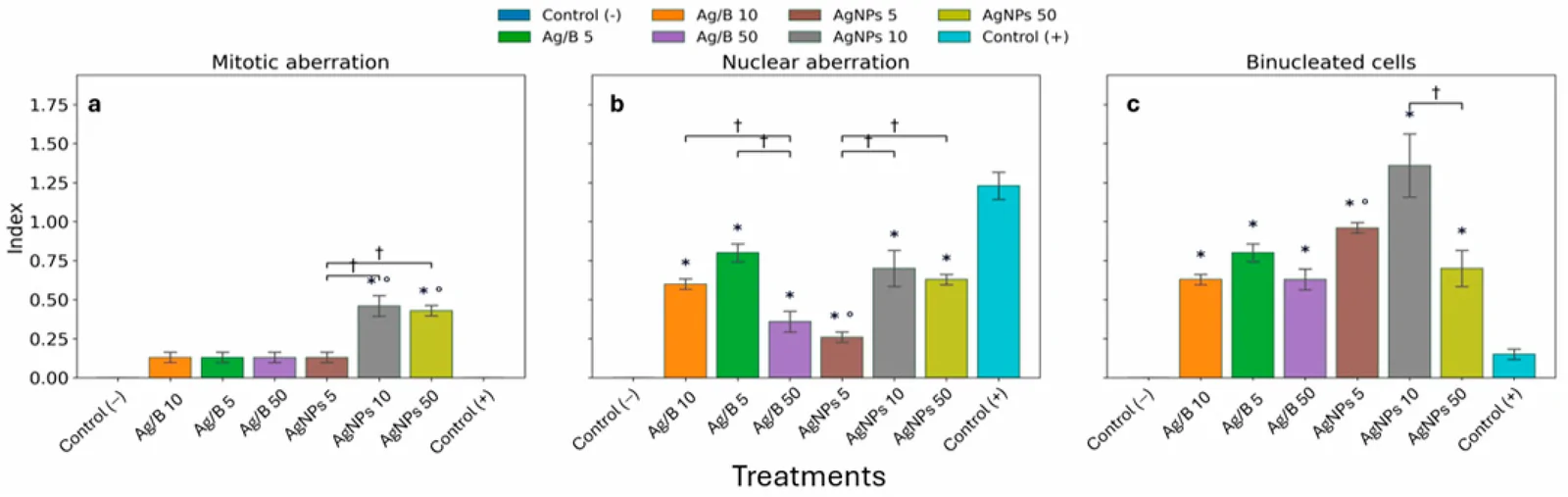
Frequency of aberrations (**a**), nuclear aberrations (nuclear buds) (**b**), and binucleated cells (**c**) in *V. faba* root tip meristems. cells after 48 h of treatment with Ag/B and AgNPs at concentrations of 5, 10, and 50 mg/L. Bars represent the mean ± standard error (SE) of three independent replicates. * indicates a significant difference compared with the negative control (*p* < 0.05). † indicate significant differences among concentrations within the same material (Ag/B or AgNPs) (*p* < 0.05). ° indicates a significant difference between bulk silver and AgNPs at the corresponding concentration (*p* < 0.05).

Mitotic aberrations (Figure 4) were evaluated as an indicator of chromosomal structural instability, reflecting changes in chromosome organization and segregation during cell division. ANOVA demonstrated a significant treatment effect (F = 21.37, *p* < 0.001). Dunnett test indicated that only AgNPs 10 and AgNPs 50 significantly increased the frequency of mitotic aberrations in comparison to the control (*p* < 0.05), while the remaining treatments exhibited no significant differences.

No significant differences were observed among bulk silver concentrations (F = 0.00, *p* = 1.000), whereas silver nanoparticles demonstrated a concentration-dependent effect (F = 15.17, *p* = 0.005), with AgNPs 10 and AgNPs 50 displaying higher values compared to AgNPs 5 (*p* < 0.05).

Comparative analysis between bulk and nanoparticulate forms revealed significant differences for Ag/B 10 vs. AgNPs 10 (*p* = 0.047) and Ag/B 50 vs. AgNPs 50 (*p* = 0.003), whereas no significant differences were observed for Ag/B 5 vs. AgNPs 5 (*p* = 1.000).

Nuclear bud frequency (Figure 4) was used as an indicator of genomic instability linked to the removal of amplified or damaged DNA during cell division. ANOVA revealed significant differences among treatments (F = 23.21, *p* < 0.001). Dunnett test showed that all silver treatments significantly elevated nuclear bud frequency compared to the control (*p* < 0.05).

Bulk silver treatments showed significant differences among concentrations (F = 16.12, *p* = 0.004), with the highest concentration (50 mg/L) showing stronger effects than 5 and 10 mg/L. Similarly, silver nanoparticles also showed significant differences (F = 10.50, *p* = 0.011), due to higher values in AgNPs 10 and AgNPs 50 compared to AgNPs 5.

Comparisons between silver forms showed significant differences only at 5 mg/L (Ag/B 5 vs. AgNPs 5, *p* = 0.001), while no significant differences were observed at 10 and 50 mg/L.

The frequency of binucleated cells (Figure 4) was assessed as an indicator of cytokinesis failure, thereby reflecting disruptions in the terminal stages of cell division. ANOVA indicated significant differences among the various treatments (F = 18.44, *p* < 0.001). Dunnett test demonstrated that all silver treatments significantly elevated the frequency of binucleated cells in comparison to the control (*p* < 0.05).

No significant differences were detected among bulk silver concentrations (F = 3.12, *p* = 0.118), whereas nanoparticles exhibited a concentration-dependent effect (F = 6.08, *p* = 0.036), with AgNPs 10 differing significantly from AgNPs 50.

Comparisons between silver forms showed a significant difference only at 5 mg/L (Ag/B 5 vs. AgNPs 5, *p* = 0.002), and no significant differences at 10 and 50 mg/L were observed.

**Figure 5:**
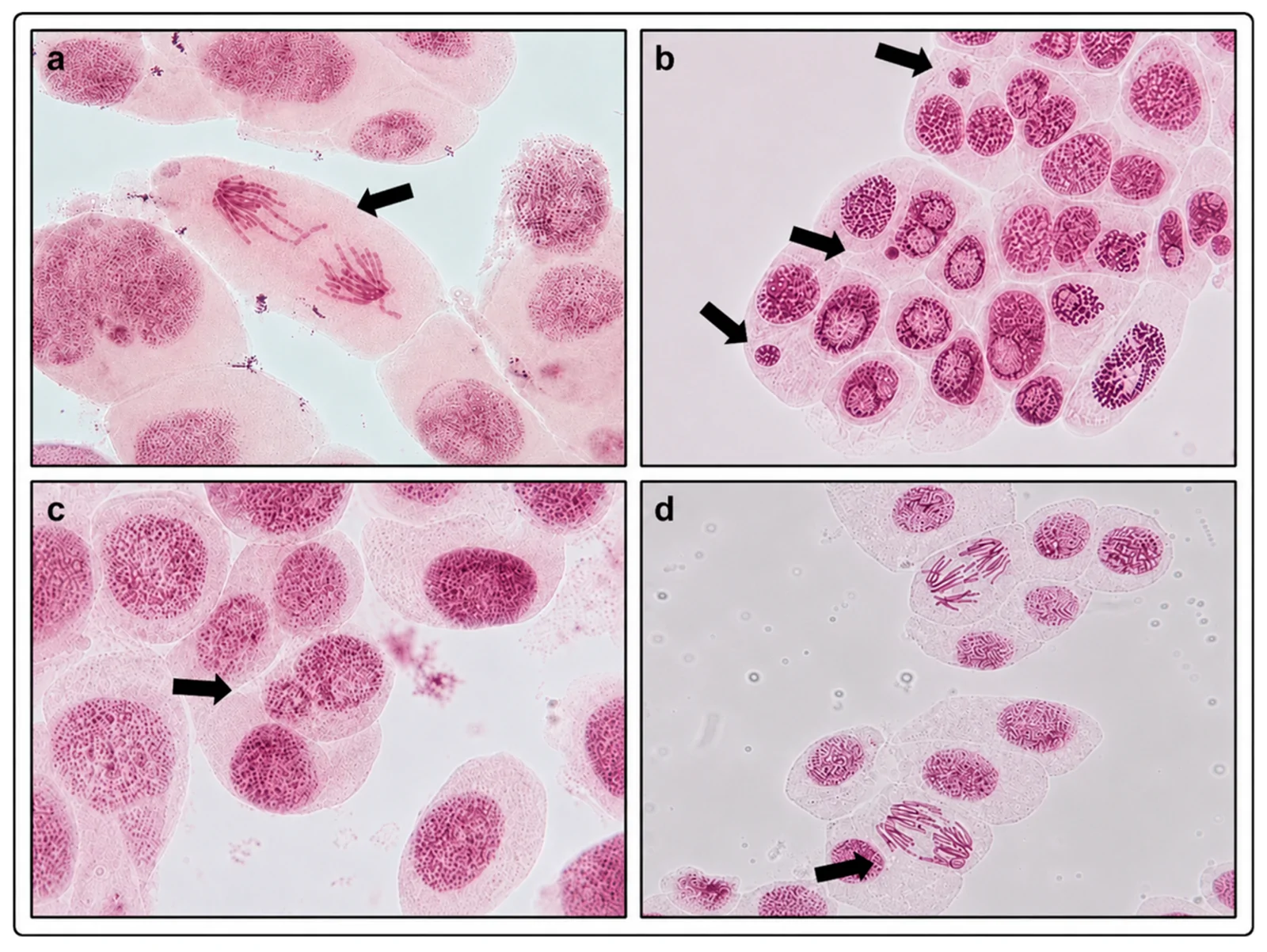
Representative cytogenetic abnormalities observed in *V. faba* root meristem cells following exposure to silver treatments: (**a**) anaphase with laggard chromosome; (**b**) micronuclei; (**c**) nuclear bud; (**d**) anaphase with a chromosome bridge.

Figure 5 shows some representative images of chromosomal aberrations.

### 2.4. Comet Assay

The comet assay was employed to assess DNA damage induced by various treatments in the root and leaf tissues of *V. faba*. Genotoxicity was evaluated through three well established parameters: tail intensity, reflecting the percentage of DNA migrated into the tail; tail length, denoting the migration extent of fragmented DNA; and tail moment, a comprehensive measure that combines the quantity of DNA in the tail with its migration distance.

#### 2.4.1. Roots Comet Assay

**Figure 6:**
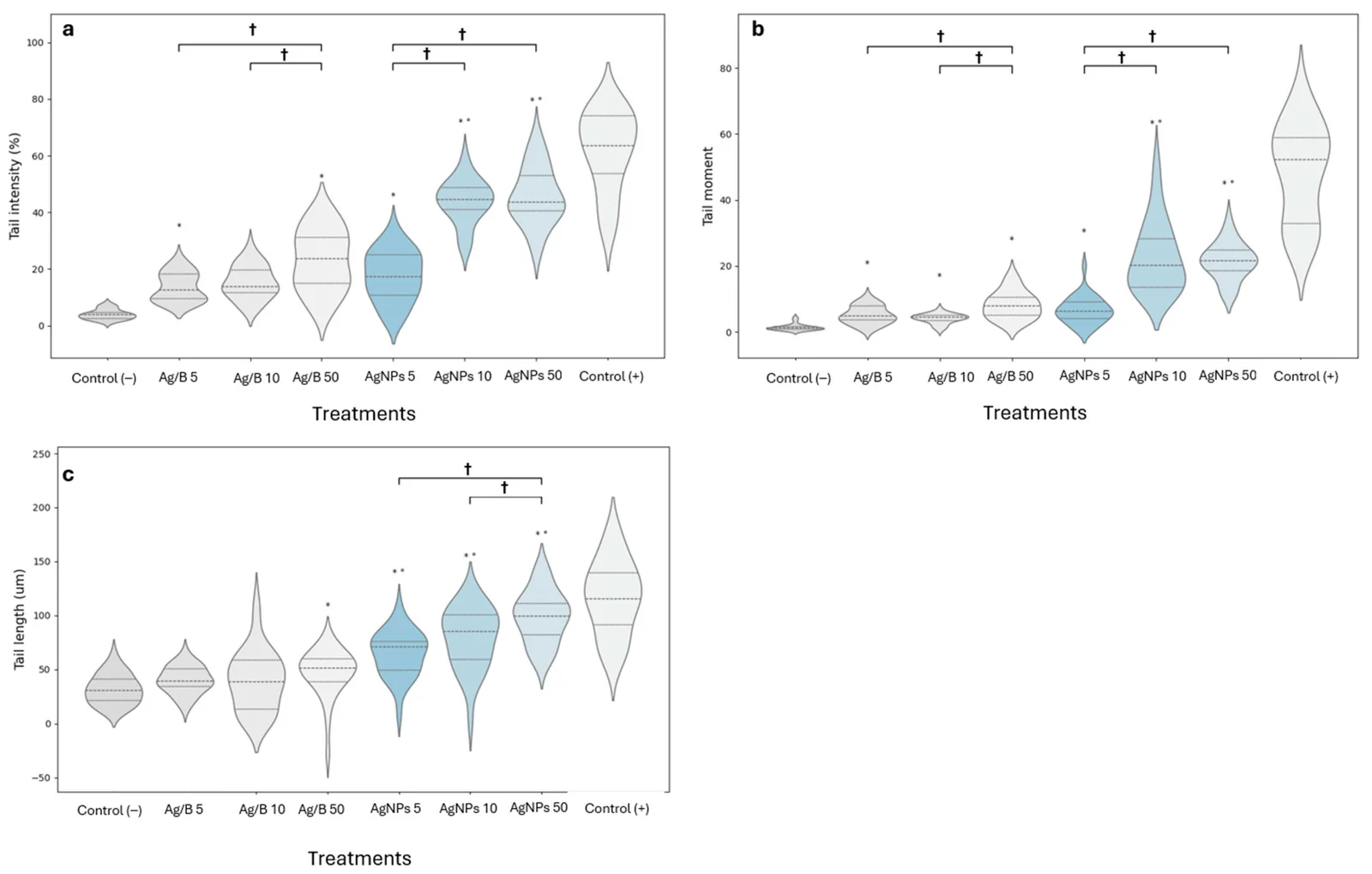
DNA damage assessment in *V. faba* root cells after 48 h of exposure to Ag/B and AgNPs using the comet assay. Violin plots represent the distribution of DNA damage parameters under each treatment condition. (**a**) Tail intensity (%), (**b**) tail moment, and (**c**) tail length (µm) was evaluated as indicators of DNA strand damage. Root cells were exposed to different concentrations of Ag/B and AgNPs (5, 10, and 50 mg/L), with negative and positive controls included for comparison. The width of each violin represents the data distribution, while internal lines indicate the median and quartile values. Increased values of comet assay parameters indicate higher levels of DNA damage. * indicates a significant difference compared with the negative control (*p* < 0.05). † indicate significant differences among concentrations within the same material (bulk silver or AgNPs) (*p* < 0.05). ° indicates a significant difference between bulk silver and AgNPs at the corresponding concentration (*p* < 0.05).

To evaluate the genotoxic potential of the treatments, DNA damage in root cells was assessed through the comet assay, utilizing tail intensity as the endpoint after 48 h of exposure. Differences among treatments were initially analyzed one-way followed by Dunnett test, employing the negative control as the reference; the results are presented in Figure 6. Subsequently, concentration-dependent effects within each silver form (Ag/B and AgNPs) were evaluated using one-way ANOVA and Tukey post hoc test. Additionally, direct comparisons between bulk silver and AgNPs at equivalent concentrations were conducted utilizing paired Student’s t-tests.

The overall ANOVA demonstrated statistically significant differences in tail intensity among the treatments (Figure 6a) (F = 81.4940, *p* < 0.05). Compared to the negative control, Ag/B significantly elevated tail intensity at concentrations of 5 and 50 mg/L (*p* < 0.05), whereas no statistically significant effect was observed at 10 mg/L (*p* = 0.1970). Conversely, all AgNPs treatments yielded significantly higher tail intensity values than the negative control (*p* < 0.05).

Within the Ag/B treatments, tail intensity was significantly greater at 50 mg/L than at 5 and 10 mg/L (F = 14.9795, *p* < 0.05), with no differences between the two lower concentrations. Similarly, AgNPs displayed a notable concentration effect (F = 44.8721, *p* < 0.05); tail intensity at 5 mg/L was markedly lower than that at 10 and 50 mg/L, whereas no differences were observed between the latter concentrations. Comparisons between the two forms of silver revealed no significant differences at 5 mg/L (*p* = 0.1353). Nonetheless, AgNPs elicited significantly higher tail intensity values than Ag/B at 10 and 50 mg/L (*p* < 0.05).

Concerning the tail moment (Figure 6b), the comprehensive ANOVA demonstrated statistically significant differences in tail moment among the treatment groups (F = 126.4923, *p* < 0.05). In comparison to the negative control, all Ag/B and AgNPs treatments significantly elevated tail moment values (*p* < 0.05).

Within the Ag/B treatments, the tail moment was markedly higher at 50 mg/L compared to 5 and 10 mg/L (F = 13.2964, *p* < 0.05), while no significant differences were identified between the two lower concentrations. Similarly, AgNPs demonstrated a notable concentration-dependent effect (F = 94.7736, *p* < 0.05), with the tail moment at 5 mg/L being significantly lower than those at 10 and 50 mg/L, whereas no differences were observed between these higher concentrations. Comparative analysis between the two forms of silver indicated no statistically significant difference at 5 mg/L (*p* = 0.0678); however, AgNPs elicited significantly higher tail moment values than Ag/B at 10 and 50 mg/L (*p* < 0.05).

Regarding tail length (Figure 6c), the variations among treatments were assessed through a one-way ANOVA followed by Dunnett test, employing the negative control as the reference standard. The comprehensive ANOVA demonstrated statistically significant differences in tail length across different treatments (F = 37.6299, *p* < 0.05). When compared to the negative control, the Ag/B treatment resulted in a statistically significant increase in tail length exclusively at 50 mg/L (*p* < 0.05), while no significant differences were observed at 5 and 10 mg/L. Conversely, all AgNPs treatments yielded tail length values that were significantly higher than those of the negative control (*p* < 0.05).

Within the Ag/B treatments, no significant concentration-dependent differences were detected (F = 1.1247, *p* = 0.3294). Conversely, AgNPs showed a significant concentration effect (F = 14.9006, *p* < 0.05), with the 50 mg/L treatment exhibiting higher tail length values than both 5 and 10 mg/L, while no significant differences were observed between the latter concentrations. Comparisons between the two silver forms revealed that AgNPs induced significantly higher tail length values than Ag/B at all tested concentrations (5, 10, and 50 mg/L; *p* < 0.05).

#### 2.4.2. Leave Comet Assay 24 Post-Treatment

**Figure 7:**
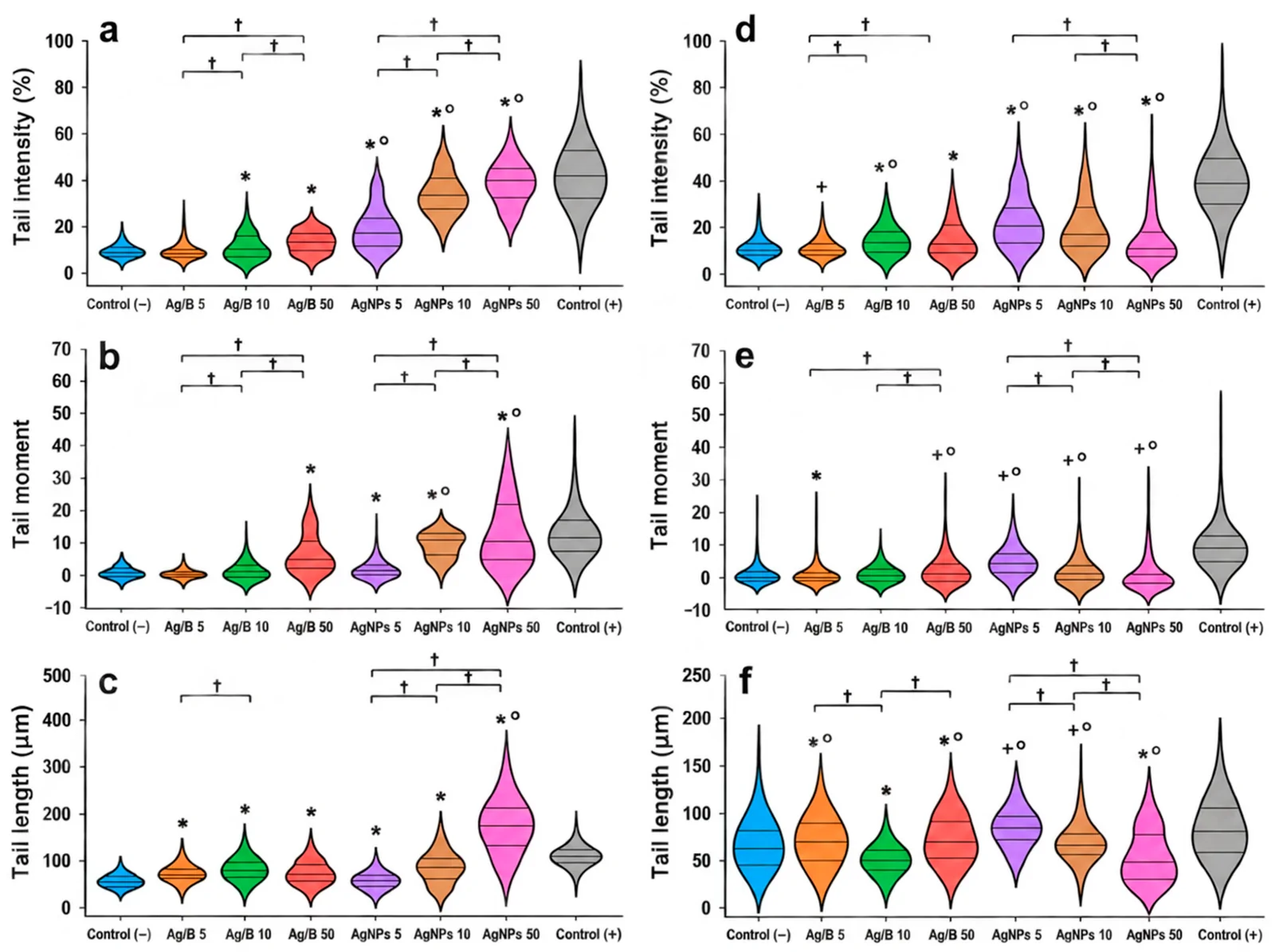
Distribution of comet assay parameters in leaf cells of *V. faba* following exposure to Ag/B and AgNPs. Panels (**a**–**c**) show tail intensity (%), tail moment, and tail length (µm) measured 24 h after treatment, whereas panels (**d**–**f**) present the same parameters measured 5 days post-treatment. Violin plots represent the distribution of the individual data points; the internal dashed lines indicate the mean, median, and interquartile range (25th and 75th percentiles). * indicates a significant difference compared with the negative control (*p* < 0.05). † indicate significant differences among concentrations within the same material (Ag/B or AgNPs) (*p* < 0.05). ° indicates a significant difference between bulk silver and AgNPs at the corresponding concentration (*p* < 0.05).

Concerning the comet assay conducted 24 h after a five day exposure to various concentrations of bulk silver and silver nanoparticles, tail intensity (refer to Figure 7a) was assessed as an indicator of DNA damage. The overall ANOVA demonstrated statistically significant differences among the treatment groups (F = 378.4437, *p* < 0.05). Compared to the negative control, Ag/B caused a notable increase in tail intensity only at concentrations of 10 and 50 mg/L (*p* < 0.05), whereas no significant effect was observed at 5 mg/L. Conversely, all silver nanoparticle treatments resulted in significantly higher tail intensity values than the negative control (*p* < 0.05).

Within the Ag/B treatments, a significant concentration-dependent effect was observed (F = 40.1456, *p* < 0.05), with significant differences among all tested concentrations. Similarly, AgNPs showed a significant concentration effect (F = 144.6927, *p* < 0.05), where all pairwise comparisons between concentrations were significantly different, indicating an increase in tail intensity with increasing AgNPs concentration. Comparisons between the two silver forms revealed that AgNPs induced significantly higher tail intensity values than Ag/B at all tested concentrations (5, 10, and 50 mg/L; *p* < 0.05).

Regarding the tail moment (Figure 7b), the ANOVA indicated significant differences in tail moment across treatments (F = 90.4148, *p* < 0.05). Relative to the negative control, Ag/B elicited a significant increase in tail moment solely at 50 mg/L (*p* < 0.05), whereas no significant effects were observed at 5 and 10 mg/L. Similarly, treatments with AgNPs significantly elevated tail moment only at 10 and 50 mg/L, with the 5 mg/L treatment showing no difference from the control (*p* = 0.4782).

Within the Ag/B treatments, a significant concentration-dependent effect was observed (F = 76.8736, *p* < 0.05), with significant differences among all tested concentrations. The tail moment increased progressively from 5 to 50 mg/L. Similarly, AgNPs exhibited a noteworthy concentration effect (F = 66.3468, *p* < 0.05), with all pairwise comparisons between concentrations indicating significant differences, thereby signifying an increase in tail moment corresponding to rising AgNPs concentrations. Comparative analysis between the two silver forms demonstrated that AgNPs elicited markedly higher tail moment values than Ag/B at all evaluated concentrations (5, 10, and 50 mg/L; *p* < 0.05).

With reference to tail length (Figure 7c), the ANOVA indicated statistically significant differences in tail length among the treatment groups (F = 160.8958, *p* < 0.05). Compared to the negative control, the Ag/B treatment notably increased tail length at all examined concentrations (5, 10, and 50 mg/L; *p* < 0.05). Conversely, the AgNPs treatments resulted in significant increases in tail length solely at concentrations of 10 and 50 mg/L, with no significant difference observed at 5 mg/L (*p* = 0.9987).

Within the Ag/B treatments, a significant concentration effect was observed (F = 6.1805, *p* < 0.05), with a significant difference only between 5 and 10 mg/L, while no differences were detected between 10 and 50 mg/L or between 5 and 50 mg/L. Similarly, AgNPs showed a significant concentration-dependent effect (F = 201.1699, *p* < 0.05), with significant differences among all tested concentrations, indicating a progressive increase in tail length with increasing AgNPs concentration. Comparisons between the two silver forms revealed that AgNPs induced significantly different tail length values than Ag/B at 5 and 50 mg/L (*p* < 0.05), whereas no significant difference was observed at 10 mg/L (*p* = 0.4695).

### 2.5. Comet Assay 5 Days Post-Treatment

DNA damage was evaluated 5 days after exposure to the bulk silver and silver nanoparticle treatments. For tail intensity (Figure 7d), the overall ANOVA showed significant differences in tail intensity among treatments (F = 23.5646, *p* < 0.05). Relative to the negative control, Ag/B increased tail intensity significantly at 10 and 50 mg/L (*p* < 0.05), whereas no significant effect was observed at 5 mg/L. In contrast, all AgNPs treatments produced significantly higher tail intensity values than the negative control (*p* < 0.05).

Within the Ag/B treatments, a significant concentration effect was observed (F = 12.4781, *p* < 0.05), with significant differences between 5 and 10 mg/L and between 5 and 50 mg/L, while no differences were detected between 10 and 50 mg/L. Similarly, AgNPs showed a significant concentration effect (F = 12.9960, *p* < 0.05), with significant differences between 50 mg/L and both 5 and 10 mg/L, whereas no difference was observed between 5 and 10 mg/L. Comparisons between the two silver forms revealed that AgNPs induced significantly higher tail intensity values than Ag/B at 5 and 10 mg/L (*p* < 0.05), while no significant difference was observed at 50 mg/L (*p* = 0.5678).

Regarding tail moment (Figure 7e), the overall ANOVA revealed significant differences in tail moment among treatments (F = 14.7997, *p* < 0.05). Relative to the negative control, Ag/B induced a significant increase in tail moment only at 50 mg/L (*p* < 0.05), whereas no significant effects were observed at 5 and 10 mg/L. In contrast, AgNPs treatments significantly increased tail moment at 5 and 10 mg/L, while no significant differences were detected at 50 mg/L.

Within the Ag/B treatments, a significant concentration effect was observed (F = 7.2899, *p* < 0.05), with higher tail moment values at 50 mg/L compared to 5 and 10 mg/L, whereas no differences were observed between the two lower concentrations. Similarly, AgNPs demonstrated a significant concentration effect (F = 16.5092, *p* < 0.05), with significant differences among all tested concentrations. Comparisons between the two silver forms revealed that AgNPs induced significantly higher tail moment values than Ag/B at 5 and 10 mg/L (*p* < 0.05), whereas no significant difference was observed at 50 mg/L (*p* = 0.0726).

Regarding tail length (Figure 7f), the overall ANOVA indicated significant differences in tail length across treatments (F = 26.6613, *p* < 0.05). Relative to the negative control, Ag/B notably increased tail length at all tested concentrations (5, 10, and 50 mg/L; *p* < 0.05). Likewise, AgNPs treatments resulted in significantly higher tail length values at 5 and 10 mg/L; however, no significant difference was observed at 50 mg/L (*p* = 0.2461).

Within the Ag/B treatments, a significant concentration effect was observed (F = 27.4181, *p* < 0.05), with significant differences between 10 mg/L and both 5 and 50 mg/L, while no differences were detected between 5 and 50 mg/L. Likewise, AgNPs showed a significant concentration-dependent effect (F = 47.0330, *p* < 0.05), with significant differences among all tested concentrations. Comparisons between the two silver forms revealed that AgNPs induced significantly different tail length values compared with Ag/B at all tested concentrations (5, 10, and 50 mg/L; *p* < 0.05).

#### Comparison of DNA Damage Parameters Between 24 h and 5 Days Post-Treatment

To assess the progression of DNA damage during the recovery phase, comet assay parameters were compared between 24 h and five days post the final exposure employing T-student (Figure 7).

About tail intensity, significant temporal changes were observed in most experimental groups. Among the bulk silver treatments, tail intensity increased significantly between 24 h and 5 days at concentrations of 5 mg/L (2.99% vs. 5.14%; *p* < 0.001) and 10 mg/L (6.37% vs. 8.97%; *p* = 0.006), whereas no significant change was detected at 50 mg/L (*p* = 0.677). In the AgNPs treated plants, no significant difference was observed at 5 mg/L (*p* = 0.294); however, tail intensity decreased significantly at 10 mg/L (30.18% vs. 14.23%; *p* < 0.001) and 50 mg/L (35.19% vs. 8.43%; *p* < 0.001). Significant differences were also identified in both control groups, with tail intensity increasing in the negative control (3.27% vs. 5.32%; *p* < 0.001) and decreasing in the positive control (41.03% vs. 32.13%; *p* < 0.001).

Tail moment analysis revealed significant differences across evaluation times in most treatments. For bulk silver, 5 mg/L showed a notable increase (0.82 to 1.94; *p* < 0.001), while 10 mg/L remained stable (*p* = 0.458). Conversely, the 50 mg/L treatment experienced a significant decrease (6.98 to 3.68; *p* < 0.001). All AgNPs treatments exhibited marked temporal variations: an increase at 5 mg/L (2.59 to 5.92; *p* < 0.001) but decreases at 10 mg/L (9.70 to 4.11; *p* < 0.001) and 50 mg/L (13.55 to 2.47; *p* < 0.001). Additionally, significant changes were noted in the negative control (1.52 to 2.27; *p* = 0.019) and the positive control (13.21 to 10.78; *p* = 0.025).

Tail length results exhibited significant increases at concentrations of 5 mg/L (58.49 vs. 84.71 μm; *p* < 0.001) and 50 mg/L (62.15 vs. 84.47 μm; *p* < 0.001), whereas no statistically significant difference was observed at 10 mg/L (*p* = 0.108). For plants treated with AgNPs, tail length increased notably at 5 mg/L (40.61 vs. 97.90 μm; *p* < 0.001) and 10 mg/L (71.45 vs. 83.08 μm; *p* = 0.006), while a substantial decrease was recorded at 50 mg/L (149.92 vs. 66.93 μm; *p* < 0.001).

Overall, these results demonstrate that the evolution of DNA damage during the post-treatment period was both concentration- and material-dependent. Bulk silver treatments exhibited stable or increased comet parameters after five days, whereas AgNP treated plants displayed a marked reduction in DNA damage at the highest concentrations, particularly for tail intensity and tail moment, suggesting partial recovery following nanoparticle exposure.

**Figure 8:**
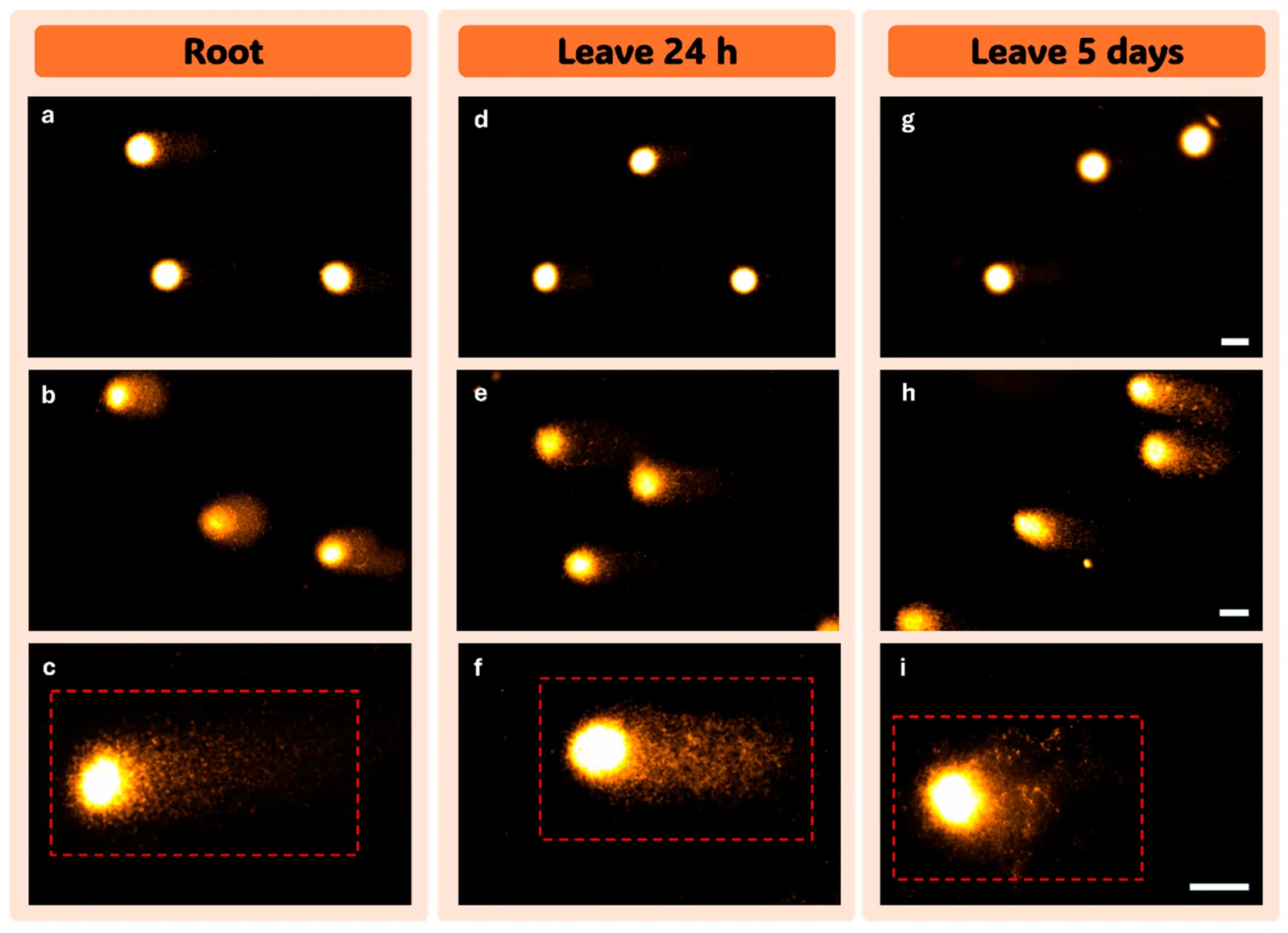
Representative comet assay images showing DNA damage in different tissues and exposure times. (**a**–**c**) Root, (**d**–**f**) leaves after 24 h, and (**g**–**i**) leaves after 5 days. Panels (**a**,**d**,**g**) correspond to the bulk treatment, whereas (**b**,**e**,**h**) correspond to AgNPs treatment. Panels (**c**,**f**,**i**) show magnified views of representative comets from the nanomaterial treatment, highlighting DNA migration associated with genotoxic damage. Magnified images were acquired at 200×. The bar represents 50 µm.

Figure 8 presents representative images of comets in root and leaf tissues.

### 2.6. Total Chlorophyll Content

**Figure 9:**
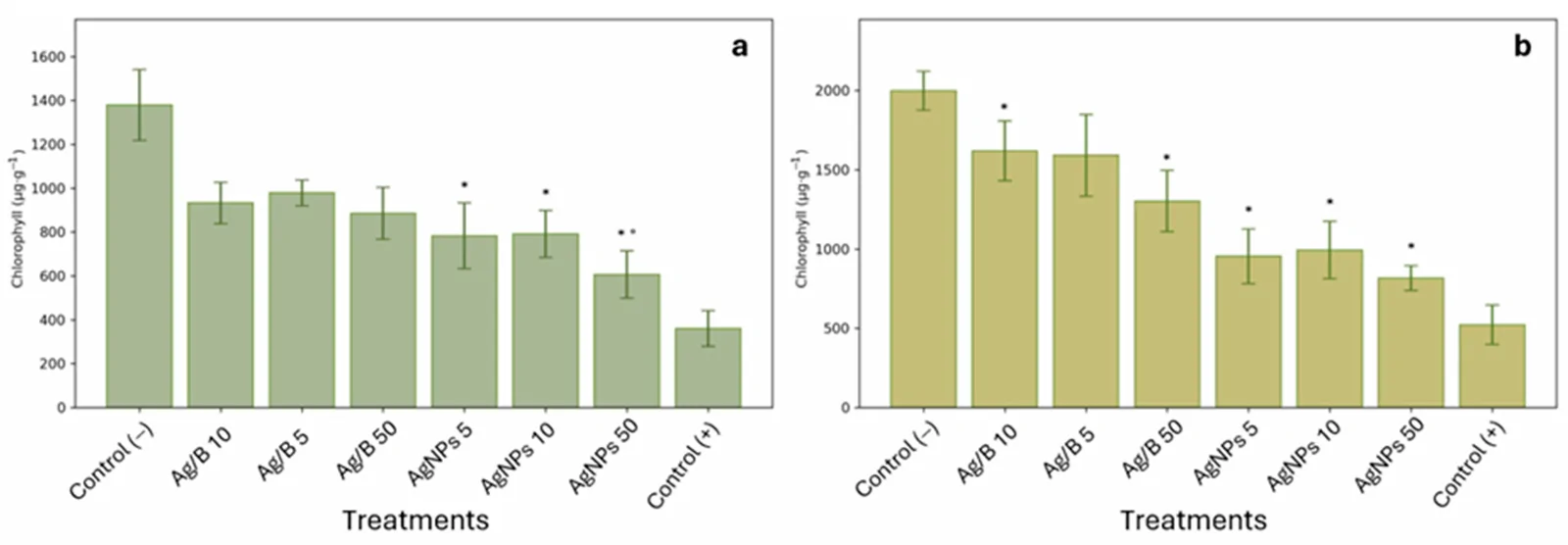
Total chlorophyll content in *V. faba* leaves following exposure to Ag/B and AgNPs. (**a**) Total chlorophyll content measured 24 h after the last treatment (24 h post-treatment). (**b**) Total chlorophyll content measured 5 days after the last treatment (5 day post-treatment). Data are presented as the mean ± standard error (SE). * indicates a significant difference compared with the negative control (*p* < 0.05). ° indicates a significant difference between bulk silver and AgNPs at the corresponding concentration (*p* < 0.05).

To assess the possible phytotoxic effects of silver bulk material and silver nanoparticles, total chlorophyll content was measured as an indicator of photosynthesis and overall plant health. These measurements started 24 h after the final treatment exposure (see Figure 9a), marking the beginning of the recovery assessment period. Monitoring continued to evaluate how different experimental groups responded during the recovery phase.

At 24 h post-treatment, a one-way analysis of variance revealed significant differences in chlorophyll content among treatments (F = 4.80, *p* = 0.007). The negative control exhibited the highest chlorophyll content (1999.3 ± 218.2), whereas all silver exposed groups presented lower mean values. According to Dunnett test, none of the bulk silver treatments differed significantly from the control (*p* > 0.05). Conversely, all AgNPs treatments significantly decreased chlorophyll content relative to the control (*p* < 0.05).

Within the Ag bulk treatments, no significant differences were observed among concentrations (F = 0.69, *p* = 0.538). Although chlorophyll content tended to decrease at 50 mg/L, Tukey post hoc test did not reveal significant pairwise differences (*p* > 0.05). Similarly, AgNPs concentrations did not differ significantly from each other (F = 0.22, *p* = 0.812), despite a decreasing trend in chlorophyll content with increasing nanoparticle concentration.

Direct comparisons between Ag/B and AgNPs treatments at equivalent concentrations showed no significant differences at 5 mg/L (t = 2.58, *p* = 0.082) or 10 mg/L (t = 1.35, *p* = 0.270). However, at 50 mg/L, plants exposed to AgNPs exhibited significantly lower chlorophyll content than those treated with Ag bulk (816.6 ± 141.4 vs. 1302.4 ± 136.0; t = 4.29, *p* = 0.023). Overall, these results demonstrate that AgNPs induced a greater reduction in chlorophyll content than bulk silver, indicating a higher phytotoxic potential of the nanoparticulate form, particularly at the highest tested concentration.

The chlorophyll content measured five days (Figure 9b) after the end of the exposure period was significantly influenced by the treatment (one-way ANOVA, F = 2.87, *p* = 0.049), suggesting that the various silver formulations elicited different responses during the recovery phase. The chlorophyll content in the negative control attained a mean value of 1379.5, whereas all treated groups demonstrated lower values. Dunnett post hoc test demonstrated that exclusively the AgNPs treated groups exhibited statistically significant reductions in comparison to the negative control. The plants subjected to AgNPs at concentrations of 10, 5, and 50 mg/L displayed mean chlorophyll contents of 791.7, 782.8, and 606.6, respectively, all of which were significantly lower than those observed in the control group (*p* < 0.05). Conversely, the treatments with bulk silver at concentrations of 10, 5, and 50 mg/L, with mean values of 978.0, 932.9, and 885.7, respectively, did not show statistically significant differences from the control, notwithstanding an observed decreasing trend.

Within each material category, no variations attributable to concentration were observed. An analysis of variance, followed by Tukey multiple comparison test, demonstrated that the chlorophyll content did not significantly differ among the three bulk silver concentrations (*p* = 0.784) or among the three AgNPs concentrations (*p* = 0.656). Similarly, direct comparisons between bulk silver and AgNPs at equivalent concentrations utilizing T-student revealed no statistically significant differences at 5 mg/L (*p* = 0.558), 10 mg/L (*p* = 0.351), or 50 mg/L (*p* = 0.163), although plants treated with AgNPs consistently exhibited lower chlorophyll levels compared to the corresponding bulk treatments.

Finally, to assess the evolution of chlorophyll content during the recovery period, total chlorophyll levels were compared between 24 h and 5 days after the last exposure for each treatment using independent-samples *t*-tests (Figure 9). The negative control exhibited a reduction in chlorophyll content over time (1999.0 vs. 1379.5 μg/L); however, this decrease was not statistically significant (*p* = 0.085). Among the bulk silver treatments, chlorophyll content decreased significantly at 5 mg/L (1620.0 vs. 932.9 μg/L; *p* = 0.032) and 50 mg/L (1302.3 vs. 885.7 μg/L; *p* = 0.039), whereas the reduction observed at 10 mg/L (1591.0 vs. 978.0 μg/L) was not statistically significant (*p* = 0.219). In contrast, no significant temporal differences in chlorophyll content were detected in any of the AgNPs treated groups, including 5 mg/L (955.0 vs. 782.8 μg/L; *p* = 0.599), 10 mg/L (994.3 vs. 791.7 μg/L; *p* = 0.538), and 50 mg/L (816.7 vs. 606.6 μg/L; *p* = 0.253).

### 2.7. Plant Growth Response

Plant growth was monitored throughout the experimental period by measuring stem elongation and normalizing the values relative to the initial plant size. The temporal growth dynamics were analyzed using a linear mixed-effects model, while logistic growth models were fitted to characterize growth kinetics under each treatment.

**Figure 10:**
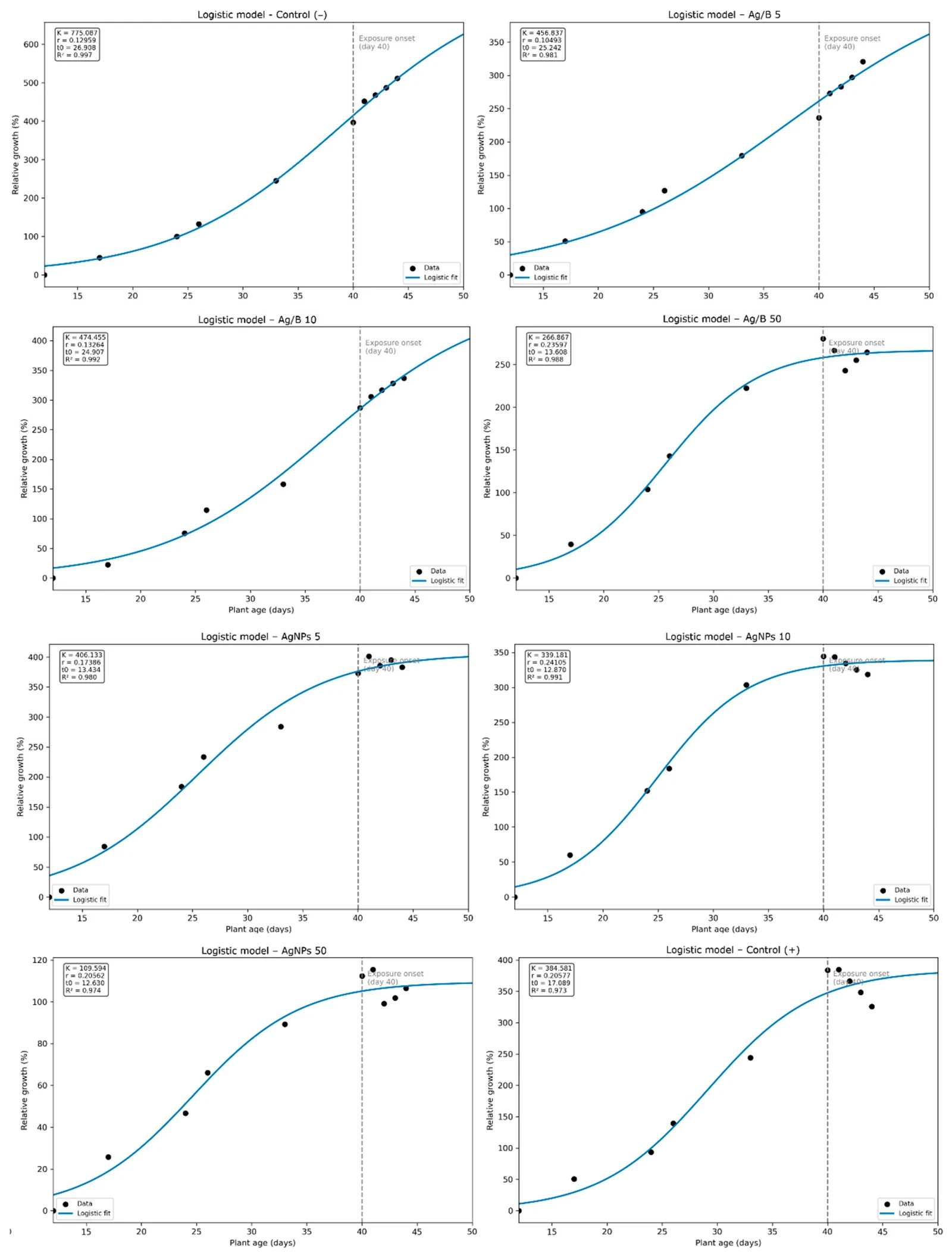
Logistic growth models of *V. faba* plants exposed to bulk silver (Ag/B) and silver nanoparticles (AgNPs). Stem elongation data were fitted using a logistic growth model for each treatment. Black circles represent the observed mean values at each sampling time, and the blue lines correspond to the fitted logistic curves. Dashed vertical lines indicate the estimated inflection point of each growth curve. Model parameters, including the carrying capacity (K), intrinsic growth rate (r), inflection point (t_0_), and coefficient of determination (R^2^), are shown within each panel. Logistic models showed excellent goodness of fit for all treatments (R^2^ = 0.973–0.997). Plants exposed to AgNPs at 50 mg/L exhibited the lowest estimated carrying capacity, indicating the greatest reduction in maximum growth potential compared with the remaining treatments.

The linear mixed effects model (Figure 10) showed a significant effect of exposure time on plant growth, with relative growth progressively increasing during the experiment. Significant increases relative to the initial measurement were detected from day 14 onward (*p* < 0.05), reflecting the normal developmental progression of *V. faba*. In contrast, no overall treatment effect was detected for either bulk silver or AgNPs at 5 or 10 mg/L. However, a significant treatment–time interaction was observed for AgNPs at 50 mg/L during the final stages of the experiment. Compared with the negative control, plants exposed to 50 mg/L AgNPs exhibited significantly lower relative growth on days 40 (*p* = 0.016), 41 (*p* = 0.008), 42 (*p* = 0.003), 43 (*p* = 0.002), and 44 (*p* = 0.001), indicating a progressive inhibition of stem elongation after repeated exposure.

Logistic growth models (Table 3) adequately described the growth trajectories of all treatments, with coefficients of determination ranging from 0.974 to 0.997, indicating an excellent fit of the experimental data. The negative control exhibited the highest estimated carrying capacity (K = 775.09), whereas AgNPs at 50 mg/L showed the lowest value (K = 109.59), corresponding to an approximately 86% reduction in maximum predicted growth. Intermediate reductions were observed for AgNPs at 5 and 10 mg/L (K = 406.13 and 339.18, respectively) and for bulk silver treatments (K = 266.87–474.45). Likewise, AgNPs treated plants reached the inflection point of the logistic curve considerably earlier (t_0_ = 24.63–25.43 days) than the negative control (t_0_ = 38.91 days), indicating an earlier slowdown in growth.

The analysis of integrated growth parameters further supported these observations. The negative control exhibited an area under the growth curve (AUC) of 6280.83 ± 1175.55 and a mean growth rate of 28.85 ± 5.09, whereas plants exposed to AgNPs at 50 mg/L showed the lowest values for both parameters (AUC = 2105.56 ± 1330.50; growth rate = 3.32 ± 1.58). Nevertheless, after adjustment for multiple comparisons using Dunnett test, none of the treatments differed significantly from the negative control for either AUC or mean growth rate (adjusted *p* > 0.05), although AgNPs at 50 mg/L consistently exhibited the greatest reduction among all treatments.

Overall, repeated exposure to AgNPs produced a concentration-dependent reduction in plant growth, with the strongest effect observed at 50 mg/L. This response was consistently supported by the mixed-effects model, the logistic growth analysis, and the integrated growth parameters, whereas bulk silver produced comparatively smaller changes throughout the experimental period.

## 3. Discussion

The environmental behavior, fate, and biological effects of nanomaterials are dictated by their physicochemical properties, which influence their colloidal stability, mobility, bioavailability, and interactions with biological systems [37,42,43].

In this study, the synthesized silver nanoparticles displayed physicochemical features indicative of a stable and well dispersed nanomaterial, with an average size of approximately 40 nm, mainly spherical morphology, a low polydispersity index (PDI = 0.176), a negative zeta potential (−27.9 ± 3.45 mV), and a distinctive surface SPR band between 390 and 410 nm. These parameters are commonly used to evaluate colloidal stability, dispersion, and the environmental and biological behavior of nanoparticles [44,45]. Collectively, they suggest a relatively uniform nanoparticle population with limited aggregation, potentially supporting sustained interactions with plant tissues during repeated exposure [37].

The nanoscale size of AgNPs influences their biological behavior because smaller particles have a higher surface area to volume ratio, increasing the number of reactive sites available for interactions with cellular membranes, proteins, and nucleic acids [46,47]. Numerous studies have shown that AgNPs within comparable size ranges (~20–50 nm) can induce more pronounced physiological and genotoxic effects in plants than larger particles or bulk silver. This may result from their greater reactive surface area, which can enhance ROS generation, facilitate Ag^+^ release, and disrupt essential cellular processes. Thus, the nanoscale dimensions of the AgNPs characterized here may partially explain the stronger biological responses observed compared with bulk silver [20,48,49]. In addition to particle size, colloidal stability influences nanoparticle bioavailability. Low PDI values and zeta potentials near ±30 mV favor stable dispersions, minimizing aggregation and preserving surface area for tissue interaction [44,50]. Accordingly, previous research has reported enhanced biological activity for highly stable AgNP suspensions, likely because individual nanoparticles remain more available for interaction with plant cells and silver-derived species. Conversely, aggregation can reduce nanoparticle mobility and their ability to interact with biological tissues [51,52].

The stability observed in the present study, including preservation of AgNP physicochemical characteristics across a broad pH range (3–10), indicates that the nanoparticles remained physically available, potentially favoring sustained interactions with plant tissues. This is relevant because pH, ionic strength, and organic matter can promote aggregation, reducing nanoparticle mobility and bioavailability. Previous studies have shown that AgNP colloidal stability is strongly influenced by environmental conditions, with aggregation altering their biological behavior [53,54].

The physicochemical properties identified in this study also help explain the differences between AgNPs and bulk silver. Despite having the same elemental composition, their biological behaviors differ because of their structural organization and surface area. Nanostructured silver possesses greater surface reactivity and a higher density of reactive sites, whereas bulk silver has a comparatively lower surface area [55,56]. Comparative studies have shown that AgNPs induce stronger responses in plants, including growth inhibition, mitotic alterations, oxidative stress, and genetic damage, than macroscopic forms of silver, confirming that the nanoscale dimension modifies the toxicity of this element [48,57]. Collectively, the physicochemical characteristics of the synthesized AgNPs provide a mechanistic basis for the biological responses observed in this study. Their small size, high colloidal stability, and extensive surface area likely promoted interactions with plant tissues, helping explain why the integrated cytogenetic, genotoxic, and physiological assessment revealed effects associated with nanoengineered silver that cannot be attributed solely to silver itself, but also to nanoscale properties [58].

Unlike many phytotoxicity studies that rely on single exposures and high concentrations (>100 mg/L) to induce rapid and readily detectable responses [43,58,59], the present study evaluated repeated exposures to moderate concentrations (5, 10, and 50 mg/L), allowing the assessment of cumulative responses associated with prolonged nanoparticle–plant interactions. Available evidence indicates that AgNP toxicity depends not only on concentration but also on exposure frequency, treatment duration, nanomaterial physicochemical properties, and organism susceptibility [60]. In this context, repeated exposure to moderate concentrations was sufficient to induce cytogenetic, genotoxic, and physiological alterations, demonstrating that cumulative exposure can produce biologically relevant effects at sublethal levels. The favorable physicochemical properties of AgNPs likely sustained their availability to plant tissues, affecting cell division, chromosomal stability, DNA integrity, and physiological performance.

The root is a key biological interface in plant nanomaterial interactions because AgNP physicochemical properties can produce measurable cellular responses. Owing to direct contact with the exposure medium and high proliferative activity, roots are commonly used to assess early cytotoxic and genotoxic effects of metallic nanoparticles [61,62]. In this study, root responses were assessed after 48 h, following two consecutive 24 h exposure periods, enabling the detection of early cytogenetic effects associated with AgNP exposure.

Repeated exposure caused marked root cytogenetic alterations, including reduced mitotic index and increased micronuclei, nuclear buds, binucleated cells, and mitotic abnormalities. These biomarkers reflect disturbances in cell proliferation, chromosomal stability, and genome integrity, consistent with responses associated with oxidative stress, impaired cell division, and chromosomal damage in plants [31,63]. The stronger response observed in AgNPs treated plants than in those treated with bulk silver suggests that nanoengineering modifies silver reactivity through differences in surface area, reactive sites, and cellular interactions. As with other nanoparticles, these nanoscale characteristics can influence biological responses beyond chemical composition alone [64].

The decrease in mitotic index indicates cytotoxicity in meristematic tissues and disturbances in cell-cycle progression. AgNPs can suppress mitotic activity by affecting cell-cycle regulators, disrupting spindle assembly, and inducing oxidative stress-related damage [43,65]. This reduction is consistent with previous findings and may reflect direct cytotoxicity and limited proliferation of cells with genomic alterations. AgNPs can adhere to the root epidermis, partially cross the cell wall, enter cellular compartments, undergo transformation, and release silver species that alter their biological activity [66,67].

The comet assay demonstrated that AgNPs induced DNA damage in root cells within 48 h, as indicated by increases in tail intensity, tail moment, and tail length. These parameters reflect DNA migration during electrophoresis and collectively provide evidence of early genotoxicity and genomic damage [68,69]. In the present study, AgNPs produced greater DNA damage than bulk silver, particularly at 10 and 50 mg/L, whereas bulk silver showed only moderate increases at the highest concentration. This suggests that the nanoscale dimension enhances silver capacity to induce genetic lesions beyond its chemical composition alone.

The stronger response to AgNPs is consistent with previous plant studies demonstrating that nanoparticle size can influence biological effects. For example, in *V. faba*, 20 nm gold nanoparticles (AuNPs) caused greater DNA damage, higher frequencies of chromosomal aberrations, and a more pronounced reduction in mitotic index than 5 and 40 nm AuNPs, illustrating the importance of nanoparticle size in determining genotoxicity [70]. Similarly, 20 nm AgNPs have been reported to induce greater cytogenetic and physiological changes than larger particles (50–73 nm), highlighting particle diameter as an important determinant of toxic potential [29]. Collectively, these findings support the premise that smaller particles, because of their higher surface area to volume ratio, can interact more extensively with cellular components and contribute to greater genotoxic effects.

The concentration-dependent response observed here is also consistent with previous findings in plant models, in which increasing AgNP concentrations resulted in progressively greater genotoxic effects. In *Allium cepa*, AgNP exposure increased chromosomal abnormalities while reducing the mitotic index in a concentration-dependent manner [71]. The comet assay detects primary DNA damage, including DNA strand breaks and alkali labile sites, and is therefore considered a sensitive biomarker of early genotoxic events [69,72]. Although the underlying mechanisms were not directly investigated in the present study, AgNP induced genotoxicity has been extensively associated with oxidative stress, which may generate DNA lesions detectable by the comet assay [73,74].

Repeated AgNP exposure induced primary DNA damage that may contribute to subsequent chromosomal and nuclear abnormalities in root tissues. This agrees with studies showing that comet assay detectable DNA lesions can precede micronucleus formation and other chromosomal aberrations when exposure persists or DNA damage exceeds repair capacity. These findings support a genotoxic progression model in which early DNA lesions may develop into stable chromosomal abnormalities when repair is incomplete or cells divide before genome restoration [75,76].

In accordance with this evidence, the increased cytogenetic abnormalities in AgNP-exposed roots indicate disruption of cell proliferation, chromosome segregation, and genome stability. The reduced mitotic index, together with chromosomal bridges, lagging chromosomes, fragments, micronuclei, nuclear buds, and binucleated cells, suggests that AgNP exposure affects DNA replication, mitotic progression, chromosome segregation, and cytokinesis. This pattern agrees with previous studies in plants, where mitotic inhibition accompanied by chromosomal and nuclear abnormalities has been interpreted as evidence of nanomaterial-induced genomic instability [31,77].

The decrease in mitotic index indicates an early cytotoxic response with reduced proliferative capacity in meristematic cells. Interference with DNA replication, spindle assembly, or chromosome segregation can reduce cell division through activation of DNA damage checkpoints, limiting the propagation of genetically compromised cells [78,79]. Chromosomal bridges, lagging chromosomes, fragments, micronuclei, and nuclear buds reflect defective segregation and genomic instability during mitosis and may result from structural damage or defects in the mitotic machinery. Micronuclei and nuclear buds can therefore represent downstream manifestations of genome instability [80,81]. In addition, binucleated cells suggest impaired cytokinesis and incomplete cytoplasmic division following nuclear division [82]. Overall, these biomarkers indicate that AgNPs interfere with several stages of cell division, affecting DNA integrity, chromosome separation, and cytokinesis, with potential consequences for genomic stability.

The agreement among these biomarkers supports a progressive process of genomic instability, in which DNA damage detected by the comet assay may represent an early event preceding chromosomal abnormalities during subsequent cell divisions.

Where root responses reflect cellular effects from direct AgNP contact in the growth medium, foliar assessment evaluated aerial tissues following independent nebulization exposure. This approach is environmentally relevant because plants may experience direct nanoparticle deposition from atmospheric processes, aerosols, or agricultural practices, resulting in leaf interactions without necessarily involving root uptake and internal translocation [83,84]. Evidence also indicates that AgNPs and other metallic nanoparticles can adhere to foliar surfaces and infiltrate leaf tissues through stomata, cuticular pathways, and other epidermal openings following spray application or atmospheric deposition [64,85]. Accordingly, the responses observed in leaves are more appropriately interpreted as direct consequences of foliar AgNP exposure rather than secondary effects of root uptake and internal translocation.

Foliar exposure represents a distinct nanoparticle plant interaction pathway, in which AgNPs initially contact the cuticle, epidermis, and stomata. Their fate is largely governed by physicochemical properties such as particle size, surface charge, colloidal stability, and Ag^+^ release, which influence surface retention, tissue penetration, and cellular interactions, ultimately contributing to molecular, cytogenetic, and physiological responses [86,87]. Experimental evidence indicates that foliar AgNP exposure can induce oxidative stress, impair photosynthetic performance, and promote DNA damage, demonstrating that aerial tissues can be direct targets of nanoparticle toxicity [86].

In this study, foliar responses were evaluated using the comet assay for DNA damage at 24 h and 5 days after exposure, and total chlorophyll content over the same period. This design combined an early molecular endpoint with a physiological indicator across two developmental stages. Ontogenetic stage is relevant because tissue age influences cuticle composition, metabolic activity, antioxidant capacity, and DNA repair efficiency, affecting susceptibility to nanoparticle toxicity [87,88].

Foliar exposure to AgNPs increased all comet assay parameters, indicating primary DNA damage in leaf tissues. This agrees with previous observations in *Nicotiana tabacum*, where spherical AgNPs (22–25 nm) increased tail moment after 24 h at 50 and 100 mg/L. Both studies demonstrate the sensitivity of the comet assay for detecting early DNA damage following foliar AgNP exposure. In the same study, plants grown in soil containing 30 mg/L AgNPs for eight weeks also exhibited significantly increased DNA damage in leaves, suggesting that AgNP induced genotoxicity can occur across different exposure scenarios and durations, whether through direct foliar contact or prolonged root uptake [89].

The detection of DNA damage following nebulization supports the interpretation that leaves represent an independent target tissue for AgNPs toxicity because nanoparticles can directly interact with foliar tissues and induce local genotoxic responses without requiring prior root uptake and internal translocation [85,86]. These findings further emphasize that AgNPs toxicity is influenced not only by concentration but also by exposure route, which determines nanoparticle uptake, bioavailability, translocation, and biological fate within the plant [37,87].

Comparison between 24 h and 5 days post-treatment showed a decrease in DNA damage markers, indicating fewer detectable primary lesions after exposure cessation. This agrees with studies in which comet assay damage decreases during recovery as DNA strand breaks are repaired and genomic integrity is restored. In *V. faba*, most repairs occur within hours and continues for approximately 33 h, substantially reducing DNA damage, although some cells may retain residual lesions [90]. Recent reviews likewise emphasize the value of the comet assay for evaluating the timing of DNA recovery after removal of genotoxic agents, including metals, nanoparticles, and other environmental contaminants [91]. However, the reduction in comet assay-detectable damage after 5 days indicates only partial recovery from primary DNA lesions and does not necessarily demonstrate complete restoration of physiological status, because the assay predominantly detects potentially repairable damage [91,92]. Therefore, these results should be interpreted together with other biomarkers to determine the extent of cellular recovery.

This distinction among biological response levels was also evident in chlorophyll content. Although primary DNA damage decreased over time, persistent alterations in photosynthetic pigments suggest that physiological effects may continue after molecular lesions decline. Previous research similarly identifies chlorophyll reduction as a sensitive indicator of physiological alterations associated with nanoparticle interactions and impaired photosynthetic activity, particularly following exposure to AgNPs and metal oxide nanoparticles [20,93].

AgNPs can impair photosynthetic function through several mechanisms, including oxidative stress, modification of photosynthetic proteins, disruption of nutrient homeostasis, and structural damage to chloroplasts [93,94]. The temporal difference between DNA recovery and physiological alterations therefore suggests that different biological levels recover at different rates: genetic lesions may be repaired relatively rapidly, whereas restoration of cellular functions and metabolism may require more time [95,96].

Differences among growth stages further suggest that ontogenetic status influences *V. faba* sensitivity to AgNPs. Young tissues generally exhibit higher metabolic activity, active cell division, and greater resource requirements, potentially increasing their susceptibility to stressors. In contrast, more mature tissues show greater differentiation and established physiological regulation, which may support stronger tolerance responses [97,98]. Thus, developmental stage may influence both AgNP uptake and the capacity of tissues to activate defense mechanisms against nanoinduced stress.

Finally, plant growth inhibition represents an integrated physiological manifestation of nanoparticle phytotoxicity, reflecting the cumulative effects of impaired cell division, DNA damage, chlorophyll depletion, and altered metabolism during exposure [60,99]. In the present study, only AgNPs at 50 mg/L significantly reduced stem elongation, suggesting that *V. faba* was able to compensate for damage induced at lower concentrations until cumulative stress exceeded its adaptive capacity. This response is consistent with studies in *Arabidopsis thaliana*, *Lolium multiflorum*, and *Allium cepa*, in which AgNP exposure produced concentration-dependent growth reductions associated with impaired cell division, oxidative stress, and decreased photosynthetic performance [43,99,100].

The logistic growth analysis further supported these findings, showing that AgNP-treated plants exhibited lower carrying capacities and reached the inflection point earlier than the negative control, indicating a premature slowdown in development. Although differences in area under the growth curve and mean growth rate were not statistically significant after correction for multiple comparisons, these parameters suggest that repeated AgNP exposure altered the overall growth trajectory. This reduction is consistent with the decreased mitotic activity, increased DNA damage, and chlorophyll depletion observed in this study, which may restrict cell proliferation and carbon assimilation required for sustained development [36,43]. Likewise, the comparatively weaker response to bulk silver agrees with studies demonstrating that AgNPs generally exhibit greater phytotoxicity than larger silver particles because of their higher bioavailability, cellular uptake, and surface reactivity [58,100]. Collectively, these findings indicate that growth inhibition represents an integrated outcome of cumulative cytogenetic and physiological damage induced by repeated AgNP exposure.

Overall, comparison of roots and leaves demonstrates that AgNP toxicity depends on exposure pathway, tissue-specific characteristics, and recovery capacity. Roots exposed through the growth medium exhibited cytogenetic alterations in cell proliferation and chromosomal stability, whereas leaves exposed by nebulization showed DNA damage and physiological alterations related to photosynthesis and growth. These differences highlight the need to integrate multiple tissues, exposure routes, and levels of biological organization in plant nanotoxicological assessments.

## 4. Materials and Methods

### 4.1. Synthesis of Silver Nanoparticles

Silver nanoparticles were synthesized through a chemical reduction process utilizing silver nitrate (AgNO_3_, CAS number 7761-88-8) as the silver precursor and sodium citrate (CAS number 6132-04-3) as the reducing agent. A stock solution of AgNO_3_ was prepared by dissolving 0.1 g of AgNO_3_ in 10 mL of deionized water, while the sodium citrate stock solution was formulated by dissolving 0.1 g of sodium citrate in 10 mL of deionized water. For the synthesis of nanoparticles, 1.25 mL of the AgNO_3_ stock solution was added to 25 mL of deionized water and subsequently heated to boiling under continuous magnetic stirring. Upon reaching the boiling point, 1.25 mL of the sodium citrate solution was added dropwise. The reaction mixture was maintained under constant stirring until a color transition from colorless to yellowish-brown was observed, signifying the formation of silver nanoparticles. No stabilizing agents were incorporated during the synthesis process. The colloidal suspension obtained was permitted to cool to ambient temperature and stored in amber containers in the dark at room temperature until subsequent physicochemical characterization and biological assays.

### 4.2. Nanoparticle Characterization

#### 4.2.1. Particle Size Distribution Analysis of AgNPs

The particle size distribution of the synthesized AgNPs was determined from SEM micrographs using ImageJ software (v1.52u, National Institutes of Health, Bethesda, MD, USA). Images were calibrated employing the respective scale bars, and the diameters of 300 individual nanoparticles were manually measured to procure statistically representative data. The measurements obtained were subsequently processed using Python (Python Software Foundation v3.14.7, Wilmington, DE, USA). The mean particle size, standard deviation, and frequency histograms were generated through custom scripts implemented in Python to assess the size distribution and homogeneity of the nanoparticle population. The elemental composition of the synthesized AgNPs was analyzed using energy-dispersive X-Ray spectroscopy (EDS).

#### 4.2.2. Dynamic Light Scattering and Surface Charge Analysis of AgNPs

The synthesized AgNPs colloidal properties were assessed by measuring their hydrodynamic diameter (Z-average), polydispersity index (PDI), and zeta potential (ζ-potential). These measurements were conducted with a Zetasizer Nano ZS analyzer (Malvern Panalytical, Malvern, UK) that uses a 633 nm He–Ne laser and is controlled via Zetasizer software version 7.13.

Prior to analysis, the nanoparticle suspension was homogenized through gentle inversion and subsequently diluted with deionized water to achieve an appropriate concentration for measurement. The dispersions were then sonicated for 10 min to mitigate particle agglomeration, followed by transfer to measurement cells. Samples were allowed to equilibrate at 25 °C prior to data collection. For each sample, three independent measurements were conducted, and the hydrodynamic diameter, PDI, and zeta potential were reported as means with standard deviations.

#### 4.2.3. Stability Assays and pH-Dependent UV–Visible Spectroscopy

The stability of the synthesized silver nanoparticles was assessed across various pH conditions employing UV–Visible spectroscopy. Measurements were conducted using a WPA Biowave S2100 diode array spectrophotometer (Biochrom Ltd., Cambridge, UK). Prior to analysis, all AgNPs suspensions were sonicated for 10 min to ensure homogeneous dispersion. Spectral measurements were recorded in the range of 200–800 nm to monitor the optical properties of the nanoparticles. For pH-dependent assays, the pH of the nanoparticle suspensions was adjusted from pH 3 to 10 using 0.1 M HCl or 0.3 M NaOH, added dropwise under continuous monitoring. The pH was measured using a calibrated pH meter (Ohaus Starter 3100, Ohaus Corporation, Parsippany, NJ, USA) prior to each adjustment and analysis.

### 4.3. Cytogenetic Analysis in V. faba

#### 4.3.1. Plant Material and Germination

Pesticide-free certified *V. faba* L. (var. minor) seeds were kindly supplied by the Instituto de Ciencias de la Atmósfera y Cambio Climático, Laboratory of Environmental Mutagenesis, UNAM. Seed germination took place under controlled laboratory conditions. Before germination, seeds were surface sterilized by soaking in 5% sodium hypochlorite for 5 min and then rinsed four times with distilled water to eliminate traces of disinfectant.

Following sterilization, the seeds were immersed in 500 mL of distilled water for a duration of 12 h to facilitate uniform imbibition. Subsequently, germination was initiated by positioning the seeds between two layers of moistened cotton and incubating them at 22 °C in darkness for five days. Seedlings were deemed appropriate for experimental application once the primary roots attained a length of 2–3 cm.

#### 4.3.2. Experimental Design

The assay was conducted according to Ma et al., 1995 [41]. For each treatment, five randomly selected *V. faba* seedlings were exposed to 30 mL of AgNPs suspensions at different concentrations, forming three experimental groups: group 1, 5 mg/L AgNPs; group 2, 10 mg/L AgNPs; and group 3, 50 mg/L AgNPs. In addition, three corresponding experimental groups were included using bulk silver salt (Ag/B) solutions at equivalent concentrations (Ag/B 5, 10, and 50 mg/L), prepared in distilled water and used for comparative assessment of nanoparticle-specific effects.

As a Control (−), five seedlings were incubated in 30 mL of distilled water. As a Control (+), seedlings were exposed to 30 mL of potassium dichromate (K_2_Cr_2_O_7_) at 0.5 mg/mL.

All experimental and control groups were maintained under identical conditions at 22 °C ± 1 °C in darkness. To minimize physicochemical changes in the exposure media and potential microbial growth, the solutions were renewed every 24 h using the same volumes of AgNPs suspensions and distilled water, respectively.

Exposure was conducted over 48 h, and cytogenetic data was collected only at this time point for all treatments.

#### 4.3.3. Mitotic Index, MN Test, Chromosomal Aberrations Assay, Nuclear Aberrations Assay

After 48 h of exposure to the corresponding AgNPs treatments and control solutions (negative and positive controls), five primary roots per treatment were collected by excising approximately 0.5 mm from the apical meristematic region.

Root tips were fixed and processed using the squash technique followed by 1% aceto-orcein staining. One slide was prepared from each root apex, and 1000 cells per slide were examined under a light microscope at 400× magnification in a microscope Nikon DM500. The mitotic index (MI), micronucleus frequency (MN), chromosomal aberrations (CA) in anaphase (laggard chromosome, chromosomal bridges, chromatid bridges, chromosome fragments, chromosome stickiness) and nuclear abnormalities (NA) were determined according to the following equations:

Mitotic index (MI, %):

(1) $$\mathrm{MI}=\frac{\mathrm{Number}\;\mathrm{of}\;\mathrm{cells}\;\mathrm{in}\;\mathrm{interphase}\;+\;\mathrm{prophase}\;+\;\mathrm{metaphase}\;+\;\mathrm{anaphase}\;+\;\mathrm{telophase}}{1000}\times 100$$

Micronucleus index (MN, %):

(2) $$\mathrm{MN}=\frac{\mathrm{Number}\;\mathrm{of}\;\mathrm{micronucleated}\;\mathrm{cells}}{1000}\times 100$$

Chromosomal aberration frequency (CA, %):

(3) $$\mathrm{CA}=\frac{\mathrm{Number}\;\mathrm{of}\;\mathrm{cells}\;\mathrm{with}\;\mathrm{chromosomal}\;\mathrm{aberrations}}{1000}\times 100$$

Nuclear abnormality frequency (NA, %):

(4) $$\mathrm{NA}=\frac{\mathrm{Number}\;\mathrm{of}\;\mathrm{cells}\;\mathrm{with}\;\mathrm{nuclear}\;\mathrm{abnormalities}}{1000}\times 100$$

In addition, the frequency of binucleated cells was recorded during the same cell count as an additional indicator of effect in cytokinesis.

### 4.4. Comet Assay in Root and Leaf Tissues

#### 4.4.1. Experimental Exposure and Nuclear Isolation in Roots

For the comet assay in roots, seed germination, seedling establishment, and root exposure were conducted under the same conditions outlined for the chromosomal aberration assay. Briefly, seedlings were cultivated until the primary roots attained a length of 2–3 cm and were then subjected to the corresponding AgNPs treatments in accordance with the previously detailed experimental design.

Nuclei were isolated from root tissues (five biological replicates were analyzed per treatment) under low light conditions to minimize additional DNA damage. Fresh root segments were placed in ice cold buffer and gently sectioned transversally using a prechilled scalpel to facilitate the release of nuclei into the medium.

The obtained nuclear suspension was promptly collected and subsequently combined with molten low melting point agarose (LMPA) within microcentrifuge tubes. The mixture was carefully homogenized through repeated pipetting to guarantee uniform distribution of nuclei while avoiding mechanical damage.

Microscope slides pre-coated with a basal layer of 1.0% normal-melting-point agarose (NMPA) were utilized as support for gel embedding. An aliquot of the nuclei agarose suspension was meticulously dispensed onto the pre-coated slides and covered to ensure uniform spreading. The slides were positioned on a cold surface to facilitate rapid gel solidification. Following polymerization, the coverslip was carefully removed, and a final layer of 0.5% LMPA was gently applied to encapsulate the nuclei within the gel matrix.

All slides were maintained under cold and light protected conditions until further processing in the alkaline comet assay.

#### 4.4.2. Experimental Exposure and Nuclear Isolation in Leaves

For the comet assay in leaves, upon germination, seedlings were permitted to develop until they attained an average height of approximately 30 cm. The plants were subsequently transferred to plastic rack trays and maintained under controlled photoperiod conditions (12 h light/12 h dark), with the root systems consistently supported in distilled water to ensure adequate hydration throughout the experimental duration.

Foliar exposure was conducted utilizing a controlled nebulization system within a sealed exposure chamber. AgNPs suspensions were freshly prepared at concentrations of 5, 10, and 50 mg/L immediately prior to each exposure, and sonicated for 15 min using a sonicator (CSientific CS-UB32 operating at 40 KHz) to ensure uniform dispersion. In parallel, bulk metal salt solutions at corresponding concentrations were prepared in distilled water and subjected to identical conditions. Plants exposed to distilled water served as the negative control, while potassium dichromate (K_2_Cr_2_O_7_) at 0.05 M was employed as the positive control for genotoxicity.

Foliar treatments were applied inside enclosed exposure chambers specifically developed for aerosol delivery. The chambers were fabricated from 4 mm thick poly(methyl methacrylate) (PMMA) panels with internal dimensions of 600 × 300 × 400 mm (0.072 m^3^), allowing the simultaneous exposure of two plants under controlled conditions. Environmental conditions inside the exposure chambers were monitored throughout the experiment using a CR1000 datalogger (Campbell Scientific, Logan, UT, USA) coupled to sensors for temperature, relative humidity, and atmospheric pressure. Independent chambers were assigned to each treatment to avoid cross-contamination between experimental groups. Nanoparticle suspensions were aerosolized using 115 kHz ultrasonic piezoelectric nebulizers (Shenzhen Electronics) coupled to 100 mL glass reservoirs. The nebulizers were installed on the upper section of the chamber and positioned to generate a symmetrical aerosol plume, ensuring that both plants received an equivalent dose and a uniform deposition pattern. Each exposure consisted of a 30 min nebulization period, during which the system generated droplets with an average diameter of approximately 5 µm at an output rate of 0.04 L h^−1^, producing a spray cone of approximately 20°. Plant placement within the chamber was standardized to minimize spatial variability in aerosol distribution and maximize the reproducibility of foliar exposure among treatments. After nebulization, plants remained inside the chamber for an additional 30 min to allow interaction with the aerosolized nanoparticles before being removed. This exposure protocol was repeated every 24 h for 5 consecutive days.

Leaf samples were collected at 24 h and 5 days after the final exposure for comet assay analysis, with the purpose of assessing both immediate DNA damage and delayed genotoxic effects related to nanoparticle exposure.

Nuclei isolation from leaf samples was conducted on each slide using a specified leaf area of approximately 2 cm^2^, obtained from fully expanded leaves with a lamina length of approximately 4 cm. Leaf sections measuring approximately 0.5 cm in width and 4 cm in length were excised, ensuring the apical region of the leaf was avoided.

The tissue sample was promptly transferred into 250 µL of ice-cold PBS and subjected to mechanical disruption using ophthalmic scissors with repeated gentle cuts to promote cell dissociation. Throughout the procedure, the samples were kept on ice in an inclined position to preserve sample integrity and reduce DNA damage prior to nuclei release.

Each sample was prepared by mixing 50 µL of nuclei suspension with 50 µL of 1% LMPA at 40 °C. The mixture was gently homogenized and 75 µL was immediately spread onto microscope slides pre-coated with 0.5% NMPA, avoiding bubble formation. Slides were allowed to solidify on a cold surface for 5 min, after which the coverslip was removed, and a final layer of 0.5% LMPA (75 µL at 37 °C) was applied to seal the preparation prior to further processing.

#### 4.4.3. Lysis, Pre-Electrophoresis, Electrophoresis, and Data Analysis

The identical procedure was employed for both root and leaf derived comet assay preparations. Following agarose embedding, the slides were subjected to incubation in cold lysis solution at 4 °C for a minimum of 1 h under darkness. The lysis solution comprised 2.5 M NaCl, 100 mM EDTA, and 10 mM Tris (pH 10), freshly supplemented with 1% Triton X-100 and 10% DMSO immediately prior to application. All subsequent steps were conducted under dark conditions to prevent additional DNA damage.

After lysis, the slides were carefully rinsed and positioned within a horizontal electrophoresis chamber filled with freshly prepared alkaline electrophoresis buffer, consisting of 300 mM NaOH and 1 mM EDTA (pH > 13, 4 °C). The slides were then equilibrated in this buffer for a duration of 15 min to facilitate DNA unwinding and the expression of alkali labile sites.

Electrophoresis was subsequently conducted for a duration of 20 min at a voltage of 25 V and a current of 300 mA under alkaline conditions, facilitating the migration of fragmented DNA toward the anode. After electrophoresis, slides were neutralized by sequential washes in 0.4 M Tris buffer, followed by fixation in absolute ethanol.

For microscopic analysis, DNA was stained with ethidium bromide at a concentration of 20 µg/mL. Comets were subsequently visualized and analyzed utilizing a fluorescence microscope Axiostar Plus (Carl Zeiss, Oberkochen, Alemania), equipped with an excitation filter of 515–560 nm and a barrier filter of 590 nm. DNA damage quantification was carried out using the Comet Assay IV software (Perceptive Instruments, Haverhill, UK), assessing standard parameters such as tail intensity, tail moment, and tail length.

### 4.5. Total Chlorophyll Content

Leaf chlorophyll content served as a physiological biomarker of phytotoxicity. It was quantified 24 h and five days after treatment. Fully expanded leaves were collected from each treatment at the specified exposure times for this analysis. Pigments were extracted with 90% acetone in dark conditions until the tissue was fully decolored. The extracts were then centrifuged to remove debris, and the supernatant was used for spectrophotometric analysis.

Absorbance was measured at 647 nm and 664.5 nm employing a UV–Vis spectrophotometer (WPA Biowave S2100 diode array spectrophotometer, Biochrom Ltd., UK). Chlorophyll concentration was determined based on the respective absorbance readings and was expressed relative to the fresh tissue weight.

Total chlorophyll content was calculated using the following equation:


Total chlorophyll = 17.9 A_647_ + 8.08 A_664.5_

This parameter was used to evaluate alterations in photosynthetic efficiency associated with silver nanoparticle exposure.

### 4.6. Foliar Growth Assessment

Stem elongation was quantified by digital image analysis using ImageJ software. A calibrated reference scale was included in each image to convert pixel measurements into centimeters. Individual plants were monitored throughout the experimental period, with three biological replicates analyzed per treatment. Silver treatments were initiated on day 40 of plant development, and stem growth was subsequently assessed relative to the initial stem length recorded immediately before exposure. Relative growth (%) was calculated for each plant according to the equation:

(5) $$\mathrm{Relative}\;\mathrm{growth}(\%)=\left(\frac{L_{t}-L_{40}}{L_{40}}\right)\times 100$$

where $L_{t}$ is the stem length at each evaluation time and $L_{40}$ is the stem length at the onset of treatment. Temporal growth responses were analyzed using linear mixed-effects models, with treatment and time as fixed effects and plant identity included as a random effect to account for repeated measurements. To further characterize growth performance, the area under the growth curve (AUC) and the average growth rate were calculated for each individual plant. In addition, nonlinear logistic growth models were fitted to the relative growth data according to the equation:

(6) $$G(t)=\frac{K}{1+e^{-r(t-t_{0})}}$$

where G(t) represents relative growth at time t, K is the asymptotic maximum growth, r is the intrinsic growth rate, and $t_{0}$ is the inflection point corresponding to the time of maximum growth acceleration. These parameters were used to quantitatively compare post-exposure growth dynamics among treatments.

### 4.7. Statistical Analysis and Image Processing

For the chromosomal aberration data, micronucleus test, comet assay, and chlorophyll content statistical analysis was performed using a one-way analysis of variance (ANOVA) to evaluate differences among treatments and the negative control, as well as to assess differences among the three AgNPs concentrations and their corresponding bulk counterparts. When significant differences were detected, post hoc comparisons were used to identify specific group differences. In addition, Student’s t-test for paired samples was applied to compare AgNPs treatments with their corresponding bulk treatments at the same concentration (5, 10, and 50 mg/L). All results were expressed as mean ± standard error, and statistical significance was considered at *p* < 0.05. Plant growth data were analyzed using a two-way analysis of variance (ANOVA), with treatment and exposure time as fixed factors. When significant differences were detected, Dunnett multiple comparison test was applied to compare each treatment with the negative control.

All statistical analyses and graphical representations were performed using Python (Python Software Foundation, Wilmington, DE, USA). Image processing and quantitative image analysis were conducted using ImageJ software (National Institutes of Health, Bethesda, MD, USA).

## 5. Conclusions

This study demonstrates that AgNPs with an average size of approximately 40 nm and high colloidal stability induced more pronounced biological effects in *Vicia faba* than bulk silver at comparable concentrations. AgNPs produced significant genotoxic and cytogenetic alterations in root tissues, including increased DNA damage, micronucleus formation, chromosomal aberrations, nuclear buds, and binucleated cells. Notably, AgNPs induced significantly greater DNA damage than bulk silver at 10 and 50 mg/L, highlighting the importance of the nanoscale form in determining the biological response to silver.

Following the cessation of exposure, DNA damage decreased in AgNP-treated plants at the higher concentrations, indicating partial recovery of primary DNA lesions. However, physiological effects persisted, as AgNPs consistently reduced chlorophyll content and exposure to 50 mg/L significantly impaired plant growth. These findings demonstrate that recovery of DNA integrity does not necessarily coincide with complete recovery of physiological function.

Overall, the results show that AgNPs exerted stronger genotoxic and phytotoxic effects than bulk silver under the experimental conditions used, with the most pronounced effects occurring at 50 mg/L. The integration of cytogenetic, genotoxic, and physiological endpoints provides evidence that nanoparticle form, concentration, exposure route, and post-exposure recovery can influence the biological response of plants to silver-based nanomaterials.

## Figures and Tables

**Table 1:** Physicochemical characterization of AgNPs determined by transmission electron microscopy (SEM), dynamic light scattering (DLS), and zeta potential analysis. Values are presented as mean ± standard deviation (SD).

Particle	SEM Diameter (nm)	Hydrodynamic Diameter (nm)	Polydispersity Index (PDI)	Zeta Potential (mV)
**AgNPs**	31.31 ± 7.53	50.94 ± 0.126	0.176 ± 0.004	−27.9 ± 3.45

**Table 2:** **Distribution of cell cycle phases and mitotic index in *V. faba* root tip cells under different treatments.** Values represent pooled numbers of examined and dividing cells across treatments in *V. faba*. For each cell cycle phase, data are presented as total pooled counts, followed in parentheses by the mean value and the corresponding percentage relative to the total number of cells analyzed per treatment. The mitotic index is expressed as mean ± standard deviation. * indicates a significant difference compared with the negative control (*p* < 0.05).

Treatment	Concentration	Pooled No. of Examined Cells	Pooled No. of Dividing Cells	Interphase	Prophase	Metaphase	Anaphase	Telophase	Mitotic Index
Control (−)	-	3000	1170	1830 (610.0; 61.0%)	1042.8 (347.6; 89.1%)	81.0 (27.0; 6.9%)	28.8 (9.6; 2.5%)	16.8 (5.6; 1.4%)	38.92 ± 4.25
Ag/B	5 mg/L	3000	534	2466.9 (822.3; 82.2%)	327.0 (109.0; 61.2%)	96.0 (32.0; 18.0%)	48.0 (16.0; 9.0%)	61.8 (20.6; 11.6%)	17.76 ± 1.94 *
	10 mg/L	3000	465	2535.9 (845.3; 84.5%)	297.9 (99.3; 64.4%)	93.9 (31.3; 20.3%)	57.0 (19.0; 12.3%)	15.0 (5.0; 3.2%)	15.46 ± 2.96 *
	50 mg/L	3000	549	2451.9 (817.3; 81.7%)	328.8 (109.6; 60.0%)	100.8 (33.6; 18.4%)	57.9 (19.3; 10.6%)	60.0 (20.0; 10.9%)	18.26 ± 1.86 *
AgNPs	5 mg/L	3000	570	2431.8 (810.6; 81.1%)	345.0 (115.0; 60.5%)	84.9 (28.3; 14.9%)	60.9 (20.3; 10.7%)	76.8 (25.6; 13.5%)	18.93 ± 1.67 *
	10 mg/L	3000	729	2271.0 (757.0; 75.7%)	471.0 (157.0; 65.7%)	117.0 (39.0; 16.3%)	108.9 (36.3; 15.2%)	31.8 (10.6; 4.4%)	24.3 ± 2.55 *
	50 mg/L	3000	891	2110.8 (703.6; 70.4%)	535.8 (178.6; 60.1%)	132.0 (44.0; 14.8%)	186.0 (62.0; 20.9%)	34.8 (11.6; 3.9%)	29.63 ± 5.12 *
Control (+)	500 mg/L	3000	102	2898 (966.0; 96.6%)	102.0 (34.0; 100%)	0 (0.0; 0%)	0 (0.0; 0%)	0 (0.0; 0%)	3.6 ± 0.18 *

**Table 3:** Estimated carrying capacity (K) obtained from logistic growth models describing the growth of *V. faba* plants exposed to bulk silver (Ag/B) and silver nanoparticles (AgNPs). * indicates a significant difference compared with the negative control (*p* < 0.05). ° indicates a significant difference between bulk silver and AgNPs at the corresponding concentration (*p* < 0.05).

Treatment (mg/L)	Control (−)	Ag/B 5	Ag/B 10	Ag/B 50	AgNPs 5	AgNPs 10	AgNPs	Control (+)
K (Maximum growth capacity)	775.09	456.84	474.45	266.87 *	406.13	339.18 *°	109.59 *°	384.58

## Data Availability

The raw data supporting the conclusions of this article will be made available by the authors on request.

## Abbreviations

The following abbreviations are used in this manuscript:

AgNPs	Silver nanoparticles
Ag/B	Silver bulk
SEM	Scanning Electron Microscopy
EDS	Energy-Dispersive X-Ray Spectroscopy
DLS	Dynamic light scattering
PDI	Polydispersity index
SPR	Surface plasmon resonance
ANOVA	One-way analysis of variance
MN	Micronucleus
MI	Mitotic index
LMPA	Low melting point agarose
NMPA	Normal melting point agarose
